# *aristaless-like homeobox-3* is wound induced and promotes a low-Wnt environment required for planarian head regeneration

**DOI:** 10.1242/dev.201777

**Published:** 2023-09-25

**Authors:** Zaleena Akheralie, Tanner J. Scidmore, Bret J. Pearson

**Affiliations:** ^1^ The Hospital for Sick Children, Program in Developmental and Stem Cell Biology, Toronto, ON M5G0A4, Canada; ^2^ University of Toronto, Department of Molecular Genetics, Toronto, ON M5S1A8, Canada

**Keywords:** Adult stem cells, Anterior regeneration, Patterning, Planarians, Wound response, Alx-3

## Abstract

The planarian *Schmidtea mediterranea* is a well-established model of adult regeneration, which is dependent on a large population of adult stem cells called neoblasts. Upon amputation, planarians undergo transcriptional wounding programs and coordinated stem cell proliferation to give rise to missing tissues. Interestingly, the Wnt signaling pathway is key to guiding what tissues are regenerated, yet less known are the transcriptional regulators that ensure proper activation and timing of signaling pathway components. Here, we have identified an aristaless-like homeobox transcription factor, *alx-3*, that is enriched in a population of putative neural-fated progenitor cells at homeostasis, and is also upregulated in stem cells and muscle cells at anterior-facing wounds upon amputation. Knockdown of *alx-3* results in failure of head regeneration and patterning defects in amputated tail fragments. *alx-3* is required for the expression of several early wound-induced genes, including the Wnt inhibitor *notum*, which is required to establish anterior polarity during regeneration. Together, these findings reveal a role for *alx-3* as an early wound-response transcriptional regulator in both muscle cells and stem cells that is required for anterior regeneration by promoting a low-Wnt environment.

## Teaser

**Summary:** *alx-3* plays an essential role in integrating wound signals, priming neurogenesis and successful head regeneration.

## INTRODUCTION

Adult multi-tissue regeneration requires the simultaneous integration of wound signals, proliferation and patterning of new tissues as they are made (Tanaka and Reddien, 2011). Tissue-resident adult stem cells (ASCs) often have the capacity to respond to wound signals and drive the compensatory proliferation that is required to remake missing tissues (Pearson and Alvarado, 2008). In addition, signaling pathways that function in specifying position and polarity in the developing embryo are often reactivated during regeneration (Gurley et al., 2010). Decades of work have gone into describing the cascades of wound responses, proliferative pathways of ASCs, and how patterning is required during various regenerative contexts, yet it is not always clear how these processes are integrated to ensure successful regeneration. In order to understand the mechanisms of adult tissue regeneration, a model system with this biological capability can yield valuable insights.

The asexual freshwater planarian *Schmidtea mediterranea* (*S. med.*) is a constitutive adult, capable of regenerating all organs after injury (Reddien and Alvarado, 2004). The remarkable regenerative abilities of planarians are reliant on a large population of cycling ASCs – called neoblasts – that constitute ∼20% of all somatic cells, some of which are pluripotent (Aboobaker, 2011; Wagner et al., 2011). Neoblasts are the only known dividing cells, are broadly distributed throughout the parenchyma and express the *piwi* homolog *smedwi-1* (hereafter referred to as *piwi-1*) (Reddien et al., 2005). Although neoblasts are molecularly heterogeneous, they are also extremely plastic in their cell fates, e.g. posteriorly located neoblasts in the tail can remake all anterior structures after amputation (Scimone et al., 2014; van Wolfswinkel et al., 2014). Thus, immense effort has been put forth on understanding the spatial patterning of both new and existing tissues during the regeneration process.

In planarians, positional control genes (PCGs) are expressed in muscle cells where they control regional identity and are required for regeneration (Witchley et al., 2013). Perhaps the most important PCG pathway in planarian is Wnt/β-catenin signaling along the anterior-posterior (AP) axis, which is required not only to maintain tissue identity as cells turnover during homeostasis but it is also the key pathway for making the polarity decision of whether a wound is anterior or posterior facing (Reddien, 2011; Sureda-Gómez et al., 2016). At homeostasis, *wnt1* is expressed at the posterior midline (posterior pole), whereas *notum*, a secreted Wnt inhibitor, is expressed at the anterior midline (anterior pole) (Petersen and Reddien, 2008, 2009a, 2011). Upon transverse injury, rapid changes in gene expression in stem cells and differentiated cells initiate regeneration (Wenemoser et al., 2012). Within 6-24 h post-amputation (HPA), the Wnt ligand *wnt1* is expressed in subepidermal cells at both anterior-facing and posterior-facing wound edges (Petersen and Reddien, 2009b), whereas *notum* is asymmetrically expressed at anterior-facing wounds (Petersen and Reddien, 2011). Sustained *wnt1* expression at posterior-facing wounds promotes tail regeneration, whereas the *notum*-dependent low-Wnt environment at anterior-facing wounds stimulates head regeneration. After wound-induced *notum* expression, a stem cell-derived population of *notum*^+^ muscle cells localizes at the anterior midline by ∼72 HPA and anterior polarity is restored (Scimone et al., 2017; Witchley et al., 2013). Disruption of Wnt signaling by RNAi of *β-catenin* can transform injury sites into ectopic heads, whereas increasing Wnt signaling with RNAi of the negative Wnt regulator *APC* will transform injury sites into ectopic tails (Gurley et al., 2008; Petersen and Reddien, 2008).

Parallel to wound patterning by PCGs, injury causes specific cascades of wound-induced gene expression, and in turn, stem cells exhibit a well characterized mitotic response to amputation (Wenemoser and Reddien, 2010; Wenemoser et al., 2012; Wurtzel et al., 2015). Interestingly, stem cells throughout the body express Wnt signal transduction effectors, including all nine planarian *frizzled* receptors (Currie et al., 2016; Molinaro and Pearson, 2016; Wurtzel et al., 2015). The sum total of these wound programs drives neoblasts to remake new tissue of the appropriate cell types. However, this raises an intriguing question: how are wound-induced programs, positional identity programs and neoblast proliferation programs integrated to ensure proper regeneration of specific structures?

In the current study, we report a role for the *aristaless-like homeobox-3* transcription factor, *alx-3*, in regulating anterior regeneration through the activation of wound-induced gene expression programs and the stem cell proliferative response. *alx-3* was initially identified as a transcription factor enriched in a population of putative neural-fated neoblasts at homeostasis (Molinaro and Pearson, 2016). We find that, in uninjured animals, *alx-3* is necessary for the maintenance of specific neuronal subtypes, including dopaminergic and GABAergic neurons, as well as for regulating brain size in proportion to body size. Surprisingly, we find that *alx-3* expression is injury induced in neoblasts and muscle cells at anterior-facing wounds by 24 HPA. Animals where *alx-3* has been knocked down by RNAi exhibit loss of anterior blastema formation and head regeneration in tail fragments. Interestingly, under conditions where the AP axis is not disrupted, *alx-3(RNAi)* animals are able to regenerate anterior tissues laterally. To explain the headless phenotype in *alx-3(RNAi)* tails, we find that *alx-3* is required for anterior *notum* expression after 6 HPA and *alx-3* expression itself is negatively regulated by Wnt signaling in a feedback loop. We conclude that *alx-3* plays an essential role in integrating wound signals and executing specific responses from anterior-localized stem cells, which subsequently primes the region for neurogenesis and successful head regeneration.

## RESULTS

### *alx-3* is expressed in putative neural-fated progenitor cells and differentiated neural cells at homeostasis

The *aristaless-like homeobox* transcription factor *alx-3* was initially identified in a single-cell transcriptomics study aimed at determining genes enriched in a putative population of neural-fated stem/progenitor cells in the homeostatic planarian head (Molinaro and Pearson, 2016). In this study, 96 single stem cells were isolated from the head region of the animal and were transcriptionally profiled. Interestingly, a gene signature was found that had stem cell gene expression, but also expressed known neural genes, which we termed nu (ν)-neoblasts (Molinaro and Pearson, 2016). From this, we identified six transcription factors enriched in the population of ν-neoblasts, of which *alx-3* was a marker, but not yet functionally examined.

**Fig. 1.**
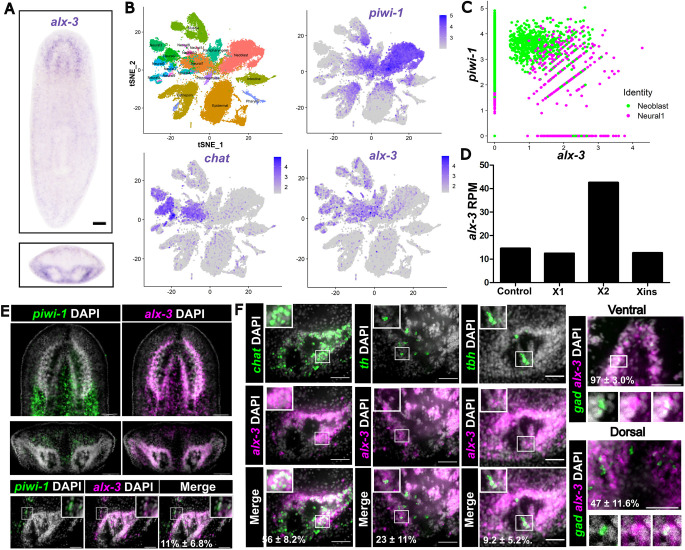
***alx-3* is a homeobox transcription factor expressed in putative neural-fated *piwi-1*^+^ cells and differentiated neural cell types at homeostasis.** (A) Whole-mount *in situ* hybridization of *alx-3* expression in a wild-type planarian imaged ventrally and cross-section through the brain. Scale bar: 100 µm. (B) tSNE representation of single-cell RNA sequencing of whole worms and clusters annotated by known cell types. Detected expression of individual genes is shown as a heatmap with gene levels in purple. Generated using data from Fincher et al. (2018). (C) Co-expression plot of *alx-3* and *piwi-1* expression from previously published single-cell RNA-sequencing data. Green dots are in the annotated neoblast cluster; magenta represents the annotated neural lineage clusters. Generated using data from Fincher et al. (2018). (D) Bar graph of *alx-3* expression in published bulk RNAseq from: whole-animal (control); FACS isolated neoblasts (X1) and progeny cells (X2); and whole-irradiated animals (Xins). (E) Fluorescence *in situ* hybridization showing *alx-3* expression with *piwi-1* (stem cells) in whole animal (top) and cross-sections through the brain (bottom). Scale bars: 100 µm. (F) Fluorescence *in situ* hybridization of brain lobe cross-sections showing *alx-3* expression with neuronal markers, including *chat* (cholinergic neurons), *th* (dopaminergic neurons), *tbh* (octopaminergic neurons) and *gad* (GABAergic neurons). Scale bars: 100 µm.

We first determined the wild-type homeostatic expression of *alx-3* using whole-mount *in situ* hybridization and observed that *alx-3* displayed strong expression in the brain along with dispersed body-wide expression (Molinaro and Pearson, 2016) (Fig. 1A). Published atlases of single-cell RNA-sequencing (scRNAseq) data reveal that *alx-3* is detected in three main cell lineages: a subset of *piwi-1^+^* stem cells; most neural cell clusters; and muscle cells (Fincher et al., 2018; Plass et al., 2018) (Fig. 1B). Further analysis of the *alx-3* and *piwi-1* co-expressing cells showed that they were annotated as ‘neural’ (Neural cluster 1), suggesting that this may be the ν-neoblast neural progenitor population (Fig. 1C). Previous bulk RNAseq data from cell populations on a flow cytometer showed expression for *alx-3* was enriched in the ‘X2’ gate, which is associated with a mix of differentiating progenitor cells and G0/G1 neoblasts (Fig. 1D) (Hayashi et al., 2006; Labbé et al., 2012; Zhu et al., 2015). Double fluorescence *in situ* hybridization of *alx-3* and *piwi-1* validated co-expression of *alx-3* in stem cells adjacent to the brain lobes at homeostasis (11±6.8% of *piwi-1*+ cells had detectable *alx-3*) (Fig. 1E).

To characterize the differentiated neuronal cell types that co-expressed *alx-3*, double fluorescence *in situ* hybridization (dFISH) was performed using neuronal subtype markers at homeostasis. Cholinergic neurons, labeled by the expression of the gene *choline acetyltransferase* (*chat*), are abundant in the planarian brain and a substantial proportion of cholinergic neurons expressed *alx-3* (56±8.2%) (Fig. 1F). Dopaminergic neurons marked by the expression of the gene *tyrosine hydroxylase* (*th*) are located medially with respect to the brain lobes and a smaller proportion of dopaminergic neurons were found to co-express *alx-3* (23±11%). Octopaminergic neurons, marked by the expression of the gene *tyrosine beta-hydroxylase* (*tbh*) are located within the medial region of the brain and only a small proportion of octopaminergic neurons co-expressed *alx-3* (9.2±5.2%). GABAergic neurons, labeled by the expression of *glutamic acid decarboxylase* (*gad*), are present in two spatially distinct populations: one located more ventro-medially (VM) and the other located dorso-laterally (DL) (Nishimura et al., 2008). Within the VM subgroup, virtually all GABAergic neurons co-expressed *alx-3* (97±3.0%), whereas roughly half of the DL subgroup co-expressed *alx-3* (47±12%). As many cholinergic neurons are also located in the peripheral nervous system, we determined that peripheral *alx-3*^+^ cells also co-expressed *chat* and overlapped with immunostaining of the axonal marker 1H6 (Fig. S1) (Ross et al., 2015). In total, the expression data demonstrate that *alx-3* is expressed in heterogenous population of neural subtypes in the brain, as well as a subset of *piwi-1*^+^ cells.

### *alx-3* is required for the maintenance *gad^+^* and *th^+^* neuronal subtypes at homeostasis

**Fig. 2.**
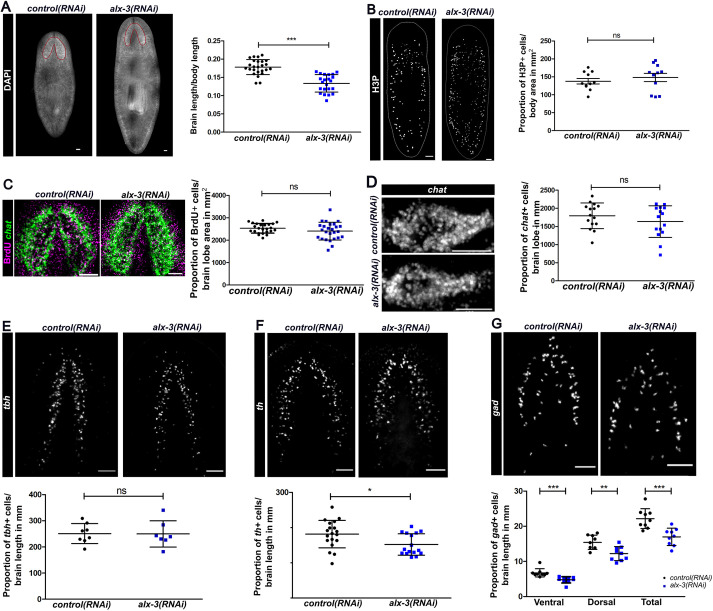
***alx-3* is required for the maintenance of *gad^+^* and *th^+^* neuronal subtypes at homeostasis.** (A) DAPI staining of whole *control(RNAi)* and *alx-3(RNAi)* animals imaged ventrally. Red line outlines the brain lobes. Quantification of length of brain lobe normalized to length of body. Data are mean±s.d., ***P<0.001. Scale bars: 100 µm. (B) Immunostaining of α-H3P in whole *control(RNAi)* and *alx-3(RNAi)* animals imaged ventrally. Quantification of the proportion of α-H3P^+^ cells normalized to the area of the animal. Data are mean±s.d. Scale bars: 100 µm. (C) Immunostaining of BrdU with fluorescence *in situ* hybridization for *chat* in *control(RNAi)* and *alx-3(RNAi)* planarians following a BrdU pulse and 7-day chase period. Quantification of the proportion of BrdU^+^ cells within the brain lobes (area outlined by *chat* expression). Data are mean±s.d. Scale bars: 100 µm. (D) Fluorescence *in situ* hybridization of *chat* expression in *control(RNAi)* and *alx-3(RNAi)* animals. Worms were cross-sectioned through the brain. Quantification of the proportion of *chat^+^* cells in one brain lobe normalized to the length of the brain lobe. Data are mean±s.d. Scale bars: 100 µm. (E) Fluorescence *in situ* hybridization of *tbh* expression in *control(RNAi)* and *alx-3(RNAi)* animals. Worms were cross-sectioned through the brain. Quantification of the proportion of *tbh^+^* cells in one brain lobe normalized to the length of the brain lobe. Data are mean±s.d. Scale bars: 100 µm. (F) Fluorescence *in situ* hybridization of *th* expression in *control(RNAi)* and *alx-3(RNAi)* animals. Worms were cross-sectioned through the brain. Quantification of the proportion of *th^+^* cells in one brain lobe normalized to the length of the brain lobe. Data are mean±s.d., *P<0.05. Scale bars: 100 µm. (G) Fluorescence *in situ* hybridization of *gad* expression in *control(RNAi)* and *alx-3(RNAi)* animals. Worms were cross-sectioned through the brain. Quantification of the proportion of *gad^+^* cells in one brain lobe normalized to the length of the brain lobe. Data are mean±s.d., **P<0.01, ***P<0.001. Scale bars: 100 µm.

To determine the functional role of *alx-3*, we assessed changes to brain morphology and neuronal populations in uninjured animals. The effect of *alx-3* knockdown on brain size and morphology was determined using DAPI staining and measuring brain length with normalization to the length of each individual animal. Although *alx-3(RNAi)* animals were generally larger in size after nine RNAi feeds, the ratio of brain length to body length was smaller compared with control animals, indicating a significant reduction in overall brain size (Fig. 2A). To determine whether reduced brain size was a result of dysregulated cell proliferation, immunostaining with anti-phosphohistone H3 (H3P) was performed in uninjured animals, labelling cells in the G2/M phase of the cell cycle. H3P staining showed no significant difference in cell proliferation levels between the *control* and *alx-3* knockdown animals (Fig. 2B). To further support this finding, homeostatic worms were fed the thymidine analogue bromodeoxyuridine (BrdU) and fixed after a 7-day chase period. To determine whether BrdU was actively incorporated into new cells in the brain lobes, BrdU^+^ cells were quantified within the region of the brain lobes defined by the expression of the neural marker *chat*, revealing no significant difference in BrdU incorporation between *alx-3* knockdown and control animals during the 7 days examined (Fig. 2C).

As *alx-3* was expressed in several neuronal populations at homeostasis, expression of each neuronal marker was assessed under *alx-3* knockdown conditions without injury. There was no substantial effect on the proportion of *chat^+^* or *tbh^+^* neuronal populations when *alx-3* expression was inhibited (Fig. 2D,E). However, *alx-3(RNAi)* animals had significantly fewer dopaminergic neurons labelled by the expression of *th*, as well as fewer GABAergic neurons labelled by the expression of *gad*, observed in both VM and DL subpopulations (Fig. 2F,G). Together, these results reveal that although *alx-3* is not required to maintain overall cell proliferation and overall neural differentiation at homeostasis, it is required to maintain appropriate brain size and number of *gad^+^* and *th^+^* neurons in uninjured animals.

### *alx-3* is expressed in stem cells at anterior-facing wounds

**Fig. 3.**
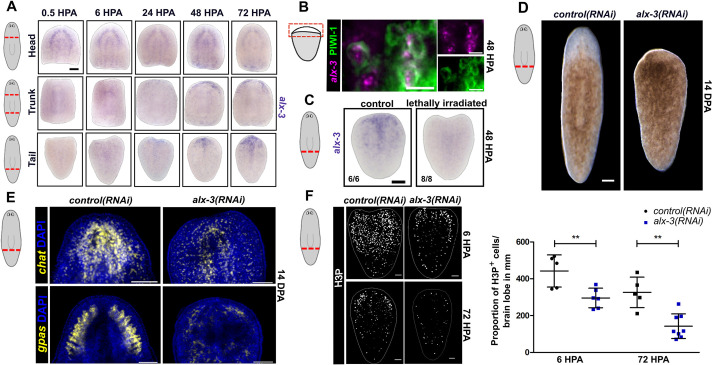
***alx-3* is expressed in stem cells at anterior-facing wounds and is required for anterior regeneration or tail fragments.** (A) Whole-mount *in situ* hybridization of *alx-3* expression in animals amputated anterior and posterior to the pharynx, and regenerated for 0.5, 6, 24, 48 and 72 HPA. Scale bar: 100 µm. (B) Double fluorescence *in situ* hybridization of *alx-3* expression and immunostaining for α-PIWI-1 in amputated tail fragments at anterior-facing wound sites at 48 HPA. Scale bars: 100 µm. (C) Whole-mount *in situ* hybridization of *alx-3* expression in control and lethally irradiated animals that were amputated post-pharynx at 48 h post-irradiation and stained at 48 HPA. Scale bar: 100 µm. (D) Live images of *control(RNAi)* and *alx-3(RNAi)* planarians after post-pharyngeal transverse amputation and regeneration for 14 days. Scale bar: 100 µm. (E) Fluorescence *in situ* hybridization of *chat* or *gpas* and DAPI staining in *control(RNAi)* and *alx-3(RNAi)* animals after post-pharyngeal transverse amputation and regeneration for 14 days. Scale bars: 100 µm. (F) Immunostaining of α-H3P in *control(RNAi)* and *alx-3(RNAi)* animals after post-pharyngeal transverse amputation and regeneration for 6 and 72 h, imaged ventrally. Quantification of the proportion of α-H3P^+^ cells normalized to length of the tail fragment. Data are mean±s.d., **P<0.01. Scale bars: 100 µm.

To determine the expression pattern of *alx-3* during regeneration, a regeneration assay was performed in which animals were amputated anterior and posterior to the pharynx, and each remaining fragment was allowed to regenerate for 0.5, 6, 24, 48 or 72 h. Whole-mount *in situ* hybridization of *alx-3* revealed that *alx-3* was detected by 24 HPA with much higher expression at anterior-facing wounds (Fig. 3A). To assess whether wound-induced *alx-3* expression was in stem cells, FISH of *alx-3* and immunostaining of PIWI-1 (using anti-PIWI-1 antibody, a kind gift from Dr Jochen Rink, Max Planck Institute for Multidisciplinary Sciences, Germany) was performed. Interestingly, *alx-3* was detected in PIWI-1^+^ cells near the anterior wound edge (Fig. 3B). To further support this finding, an irradiation assay was performed to ablate stem cells. Whole animals were lethally irradiated and amputated posterior to the pharynx at 48 h post-irradiation, tail fragments were then stained for *alx-3* expression at 48 HPA. This assay revealed that wound-induced *alx-3* expression was also ablated in the absence of stem cells (Fig. 3C). Together, these results demonstrate that *alx-3* expression is induced by injury in stem cells, primarily at anterior-facing wounds.

### *alx-3* is required for anterior regeneration in tail fragments

*alx-3* expression is induced in stem cells after injury, thus we next explored the role of *alx-3* during regeneration. After *alx-3* RNAi, animals were amputated anterior and posterior to the pharynx, and each fragment allowed to regenerate for 14 days. Knockdown of *alx-3* expression produced a noticeable phenotype where tail fragments fail to regenerate an anterior blastema and head (44% of tail fragments) (Fig. 3D). Trunk fragments, however, were consistently capable of head regeneration (Fig. S2), which is reminiscent of animals with knockdown of *follistatin* (Tewari et al., 2018). Owing to the similarity in the *alx-3(RNAi)* and *follistatin(RNAi)* phenotype, we assessed whether *follistatin* expression was impacted by *alx-3(RNAi).* Although we observed a significant reduction in the number of *follistatin^+^* cells at 48 HPA in *alx-3(RNAi)* tail fragments, overall *follistatin* expression was largely intact (Fig. S3)*.*

*alx-3(RNAi)* head and trunk fragments did not exhibit regeneration defects compared with tail fragments, despite similar levels of knockdown (Fig. S4), so we then focused on how head regeneration was disrupted in tail fragments only. Compared with controls, *alx-3(RNAi)* tail fragments that exhibited loss of head regeneration also resulted in loss of brain regeneration, as indicated by the disruption of *chat* and *gpas* fluorescence *in situ* hybridization labelling the brain lobes (Fig. 3E). *alx-3(RNAi)* tail fragments exhibited ventral nerve cords fusing at the anterior midline without regenerating brain tissue proper. As *alx-3(RNAi)* tail fragments do not regenerate a head, we wondered whether they were capable of regenerating other missing tissues. To assess this, we performed whole-mount *in situ* hybridization of the pharynx marker *foxA* (Adler et al., 2014), which revealed that when *alx-3(RNAi)* tail fragments do not regenerate a head; they also do not regenerate a pharynx (Fig. S5).

Failure in regeneration is often associated with abnormal stem cell dynamics, such as defects in cell proliferation or differentiation. To determine whether the loss of anterior regeneration may be due to dysregulated stem cell proliferation in *alx-3(RNAi)* tail fragments, cell proliferation was quantified using H3P. Planarians have two distinct waves of cell proliferation after injury, the first occurs at 6 HPA followed by a second peak at 72 HPA (Baguñà, 1976; Saló and Baguñà, 1984; Wenemoser and Reddien, 2010). Stem cell proliferation was significantly reduced during both mitotic peaks in *alx-3(RNAi)* animals (Fig. 3F). H3P staining of sagittally amputated *alx-3(RNAi)* animals did not result in a significant difference in stem cell proliferation during either mitotic peak. Similarly, the first mitotic peak in *alx-3(RNAi)* animals with incision injuries did not result in reduced stem cell proliferation (Fig. S6). As knockdown of *alx-3* in uninjured animals did not show an appreciable difference in cell proliferation, we conclude that the role of *alx-3* in injury-induced stem cell proliferation is specific to regeneration when the anteroposterior axis is disrupted.

### Failure of anterior regeneration in *alx-3(RNAi)* animals is not due to defects in neurogenesis

**Fig. 4.**
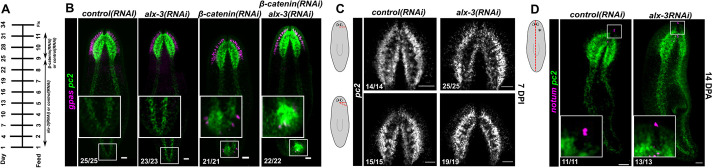
**The defect in anterior regeneration in *alx-3(RNAi)* animals is not due to defects in neurogenesis.** (A) control, *alx-3* and *β-catenin* RNAi regimen used in experiments. (B) Double fluorescence *in situ* hybridization of *gpas* and *pc2* in *control(RNAi)*, *alx-3(RNAi)*, *β-catenin(RNAi)* and double *β-catenin(RNAi)* and *alx-3(RNAi)* whole animals. Insets show tails and ectopic brains. Scale bars: 100 µm. (C) Fluorescence *in situ* hybridization of *pc2* in *control(RNAi)* and *alx-3(RNAi)* animals after incision and wedge-cutout injuries applied below the eye, as shown in the schematic, and regenerated for 7 days. Scale bars: 100 µm. (D) Double fluorescence *in situ* hybridization of *pc2* and *notum* in *control(RNAi)* and *alx-3(RNAi)* animals after sagittal amputation, as shown in the schematic, and regenerated for 14 days. Asterisk indicates the side of animal that is regenerated. Insets show anterior pole expression of *notum*. Scale bars: 100 µm.

To determine whether failure of brain regeneration was a result of defects in neurogenesis in the absence of *alx-3*, the well-established *β-catenin(RNAi)* phenotype was used as it results in the formation of ectopic heads and brain tissue at posterior regions of the animal, even without injury (Gurley et al., 2008, 2010; Petersen and Reddien, 2008, 2009b; Reuter et al., 2015; Witchley et al., 2013). Animals were given a combination of either *control(RNAi)* or *alx-3(RNAi)* for nine feeds, followed by two feeds of either *control(RNAi)* or *β-catenin(RNAi)* (Fig. 4A)*.* After the RNAi knockdown regimen, the formation of ectopic brain tissue was assayed using fluorescence *in situ* hybridization of the neural markers *gpas* and *pc2*. In *β-catenin* and *alx-3* double RNAi conditions, animals were able to generate ectopic neural tissue at posterior regions of the animal, similar to *β-catenin(RNAi)* animals (Fig. 4B). To investigate this result further, brain regeneration after minor injuries was evaluated. An incision or wedge cut-out was made below the right eye of animals without exceeding the midline and regeneration was assessed 7 days post-injury (DPI). *alx-3(RNAi)* animals were able to regenerate brain tissue following either injury type, similar to control animals (Fig. 4C).

Brain regeneration is often contingent on anterior polarity specification after injury, thus performing sagittal amputations, which do not require re-establishment of AP polarity, allows the separation of AP position from brain regeneration. In sagittally amputated animals, fluorescence *in situ* hybridization of *pc2* revealed that the missing brain lobe in *alx-3(RNAi)* animals regenerated similar to *control* animals by 14 DPA (Fig. 4D). Fluorescence *in situ* hybridization of the anterior pole marker *notum* was performed to confirm that the anterior pole and overall AP polarity remained intact following amputation. We conclude from these experiments that *alx-3* is not required for neural regeneration and that defects in neurogenesis do not explain the headless phenotype seen in *alx-3(RNAi)* tail fragments.

### *alx-3* is required for transcriptional injury responses in tail fragments

**Fig. 5.**
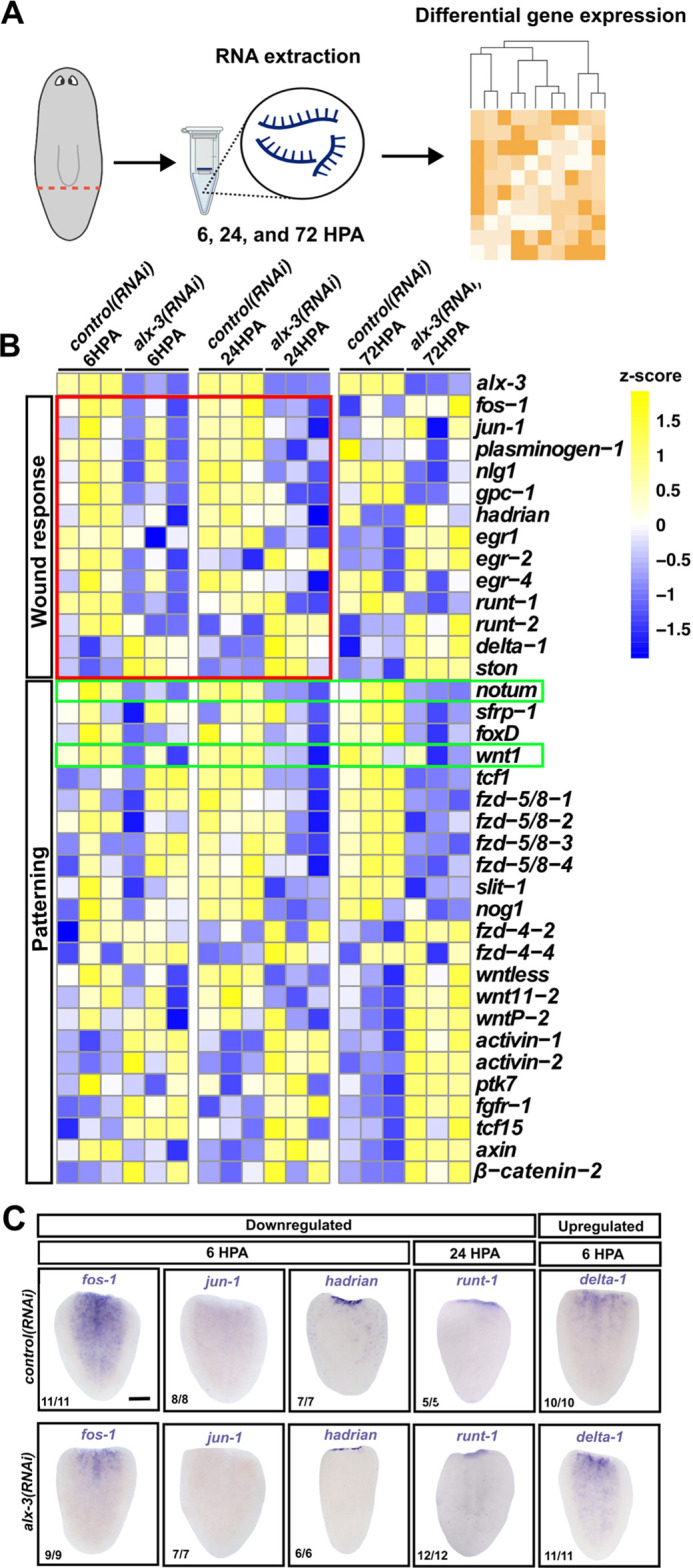
***alx-3* is required to initiate the transcriptional injury response in tail fragments.** (A) Schematic of bulk RNA-sequencing experiment. *control(RNAi)* and *alx-3(RNAi)* animals were amputated below the pharynx and regenerated for 6, 24 and 72 h before RNA extraction. (B) Heatmap of differentially expressed genes in *control(RNAi)* and *alx-3(RNAi)* tail fragments. Genes were selected based on their determined or postulated relevance to wound responses indicated by the label on the left. Replicates are displayed independently. Red box outlines early genes expressed in the first wave of gene expression in response to injury. Green boxes indicate Wnt singling genes that are injury responsive at 6-24 HPA. (C) Whole-mount *in situ* hybridization of early wound response genes found dysregulated in *alx-3(RNAi)* tail fragments identified from RNA-sequencing.

Planarian regeneration involves several tightly regulated transcriptional wound responses. The earliest wound response to occur in planarians is the transcription of early acting genes in differentiated epidermal and subepidermal cells within 30 min after injury (Wenemoser et al., 2012). These genes are rapidly transcribed as an immediate genomic response to injury and their roles may include wound closure, apoptosis, activation of downstream wound responses and stem cell proliferation (Wenemoser et al., 2012). We reasoned that dysregulated expression of genes involved in characterized wound responses in the absence of *alx-3* expression would provide insight into the functional role of *alx-3*. To this end, we performed RNAseq of *alx-3(RNAi)* or *control(RNAi)* whole-tail fragments at 6, 24 and 72 HPA, and compared differential gene expression between the conditions. (Fig. 5A). Analysis of RNAseq data between *control(RNAi)* and *alx-3(RNAi)* tail fragments identified several dysregulated wound response genes (Fig. 5B). For example, *fos-1*, *jun-1*, *plasminogen-1* and *hadrian* expression were downregulated at 6 HPA, whereas *delta-1* and *ston* expression were upregulated at 6 HPA in *alx-3(RNAi)* tail fragments. The Runx-family transcription factor *runt-1* is known to be wound-induced in stem cells and is required for daughter cell specification during regeneration (Wenemoser et al., 2012). We observed that in *alx-3(RNAi)* tail fragments, *runt-1* was downregulated at each time point. Whole-mount *in situ* hybridization staining in tail fragments between *control* and *alx-3* RNAi conditions supported the changes in gene expression observed in the RNAseq data (Fig. 5C). Ultimately, these results indicate that *alx-3* is required to activate key components of the early and late transcriptional wound responses during head regeneration in tail fragments.

To determine whether the expression of early wound-response genes is required for wound-induced *alx-3* expression, knockdown of *fos-1* was performed. Similar to previous reports by Wenemoser et al. (2012), *fos-1(RNAi)* animals produced smaller blastemas, although no other severe regeneration defects were observed at 7 DPA (Fig. S7A). *fos-1(RNAi)* resulted in slightly reduced wound-induced *notum* expression at 24 HPA as well as reduced *alx-3* expression at 48 HPA, although it was not significant enough to ablate anterior regeneration. (Fig. S7B and S7C, respectively.)

### *alx-3* is required for *Wnt* and/or *β-catenin* signaling, and for restoring anterior regeneration polarity

The Wnt signaling pathway plays a key role in the choice between anterior or posterior regeneration. Even in planarian species that cannot regenerate heads from tail fragments, if Wnt signaling is decreased, head regeneration can be restored (Liu et al., 2013; Sikes and Newmark, 2013; Umesono et al., 2013). The transcriptional injury response involves regulation of Wnt signaling genes at wound sites between 6 and 24 h post-injury (Gurley et al., 2010; Petersen and Reddien, 2009a, 2011; Wurtzel et al., 2015). Although there are many components, *wnt1* and *notum* expression direct the regeneration outcomes depending on the context of the wound. (Tewari et al., 2018; Wenemoser et al., 2012; Wurtzel et al., 2015). For example, *wnt1* is expressed at both anterior and posterior-facing wounds, whereas anterior-facing wounds asymmetrically express the Wnt inhibitor *notum* in order to achieve a low-Wnt environment required for head regeneration (Gurley et al., 2010; Petersen and Reddien, 2009a, 2011; Wenemoser et al., 2012).

**Fig. 6.**
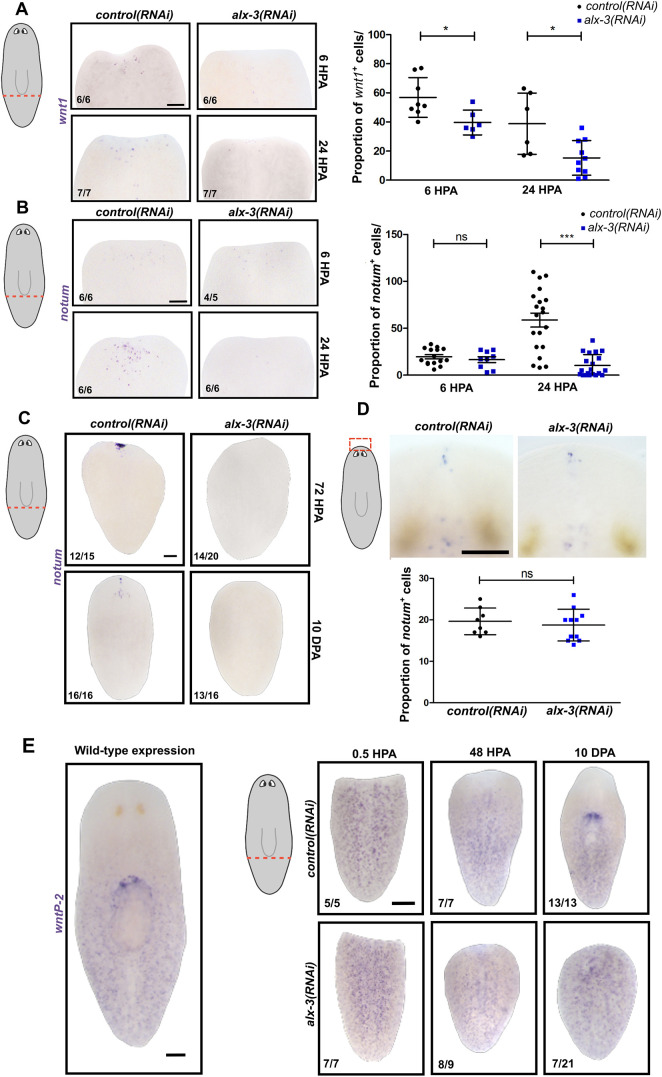
***alx-3* is required to regulate Wnt signaling during anterior regeneration.** (A) Whole-mount *in situ* hybridization of *wnt1* expression in *control(RNAi)* and *alx-3(RNAi)* amputated tail fragments at 6 and 24 HPA. Quantification of the proportion of cells that expresses *wnt1* at anterior-facing wound sites. Data are mean±s.d., *P<0.05. (B) Whole-mount *in situ* hybridization of *notum* expression in *control(RNAi)* and *alx-3(RNAi)* amputated tail fragments 6 and 24 HPA. Quantification of proportion of cells that express *notum* at anterior-facing wound sites. Data are mean±s.d., ***P<0.001. (C) Whole-mount *in situ* hybridization of *notum* expression in *control(RNAi)* and *alx-3(RNAi)* amputated tail fragments at 72 HPA and 10 DPA. (D) Whole-mount *in situ* hybridization of *notum* expression in *control(RNAi)* and *alx-3(RNAi)* uninjured animals. Quantification of the proportion of cells that expresses *notum* at the anterior pole. Data are mean±s.d. (E) Whole-mount *in situ* hybridization of *wntP-2* expression in wild-type worm (left), and *control(RNAi)* and *alx-3(RNAi)*amputated tail fragments at 0.5 HPA, 48 HPA and 10 DPA. Scale bars: 100 µm.

Consistent with dysregulation of early-acting wound response genes, RNAseq data revealed downregulation of *wnt1* and *notum* expression at all time points examined (Fig. 5B). To investigate the requirement of *alx-3* during anterior regeneration polarity, *alx-3(RNAi)* tail fragments were stained for *wnt1* and *notum* expression. A significant reduction in the number of *wnt1*-expressing cells at anterior-facing wounds was observed in *alx-3(RNAi)* tail fragments compared with the *control(RNAi)* at 6 HPA (Fig. 6A). Interestingly, the reduction in *wnt1* expression was not observed at either anterior or posterior-facing wounds of trunk fragments (Fig. S8A). Although *wnt1* is expressed broadly at both anterior- and posterior-facing wounds, determining the effect of *alx-3* knockdown on *notum* expression at anterior-facing wounds was of particular importance considering the defect in anterior regeneration in *alx-3(RNAi)* animals. Whole-mount *in situ* hybridization of *notum* revealed a significant reduction in the number of *notum-*expressing cells compared with *control(RNAi)* tail fragments at 24 HPA (Fig. 6B). However, *notum* expression was also significantly reduced in trunk fragments at the same time point (Fig. S8B). These data demonstrate that *alx-3* is required for activation of *wnt1* and *notum* expression at anterior-facing wounds of tail fragments.

At 72 HPA of head regeneration, *notum* expression resolves into an anterior pole in a stem cell-dependent manner (Oderberg et al., 2017; Vogg et al., 2014). However, *alx-3(RNAi)* tail fragments did not regenerate the *notum^+^* anterior pole at 72 HPA (Fig. 6C). To determine whether anterior pole formation was delayed in *alx-3(RNAi)* tail fragments, *notum* expression was assessed at 10 DPA; similarly, absence of *notum* expression was observed (Fig. 6C). Amputated trunk fragments were able to recover their *notum*-pole expression at 10 DPA (Fig. S8C). Notably, *alx-3* was not required to maintain the population of *notum^+^* cells in uninjured animals (Fig. 6D). These data demonstrate that *alx-3* is required to regulate *wnt1* and *notum* expression during head regeneration in tail fragments.

After amputation, positional information across the remaining tissue must rescale and reform expression domains to regenerate a correctly proportioned animal (Reddien and Alvarado, 2004). For example, the Wnt ligand *wntP-2* is expressed in a posterior-to-anterior gradient and upon amputation; tail fragments initially express *wntP-2* across the entire length of the fragment and will then rescale expression over the first week of regeneration (Gurley et al., 2010). To test whether overall body-plan reorganization was affected in *alx-3* knockdown animals, whole-mount *in situ* hybridization of *wntP-2* was performed. Unlike control animals that gradually repress *wntP-2* expression from the anterior region of tail fragments between 48 HPA and 10 DPA, the expression of *wntP-2* was observed at anterior-most regions of *alx-3(RNAi)* animals and was never properly rescaled (Fig. 6E). Taken together, these data indicate that *alx-3* expression is required to regenerate anterior polarity and restore the AP axis in tail fragments.

### *alx-3* functions in a feedback loop with Wnt signaling

**Fig. 7.**
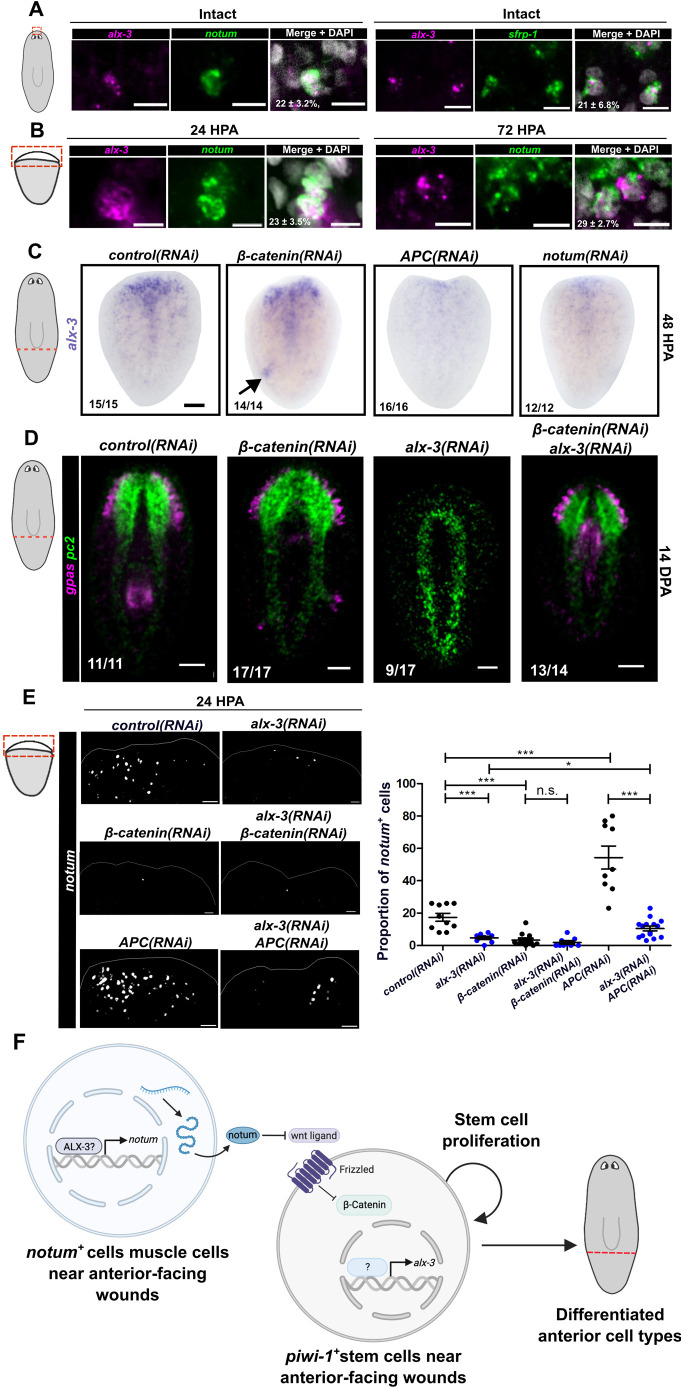
***alx-3* expression is negatively regulated by Wnt signaling.** (A) Double fluorescence *in situ* hybridization of *alx-3* and *notum* or *sfrp-1* and DAPI at the anterior pole of wild-type uninjured animals. Scale bars: 10 µm. (B) Double fluorescence *in situ* hybridization of *alx-3* and *notum*, and DAPI staining at anterior-facing wound sites in wild-type amputated tail fragments at 24 HPA and 72 HPA. Scale bars: 10 µm. (C) Whole-mount *in situ* hybridization of *alx-3* in *control(RNAi)*, *β-catenin(RNAi)*, *APC(RNAi)* or *notum(RNAi)* tail fragments 48 HPA. Scale bars: 100 µm. Arrow indicates ectopic brain in *β-catenin(RNAi).* (D) Double fluorescence *in situ* hybridization of *pc2* and *gpas* in *control(RNAi)*, *β-catenin(RNAi)*, *alx-3(RNAi)* or *β-catenin/alx-3* double RNAi*.* Scale bars: 100 µm. (E) Fluorescence *in situ* hybridization of *notum* in *control(RNAi)*, *alx-3(RNAi)*, *β-catenin(RNAi)*, double *β-catenin:alx-3(RNAi)* and double *APC:alx-3(RNAi)* tail fragments at 24 HPA. Quantification of proportion of cells that express *notum* at anterior-facing wound sites. Data are mean±s.d. *P<0.05, ***P<0.001. Scale bars: 100 µm. (F) Model of *alx-3* function in muscle cells and stem cells in tail fragments located at anterior-facing wound sites.

Although *alx-3* was wound induced in stem cells, we also observed *alx-3* expression in *notum*^+^ and *sfrp-1*^+^ pole cells in uninjured animals (22±3.2% of *notum*^+^ cells, 21±6.8% of *sfrp-1*^+^; Fig. 7A). Thus, we reasoned that *notum* expression during head regeneration may require *alx-3* cell-autonomously. We determined the dynamics of co-expression between *alx-3* and *notum* during regeneration and found that *alx-3* expression was observed in wound-induced *notum*^+^ cells at both 24 HPA (23±3.5%) and at 72 HPA (29±2.7%; Fig. 7B). Based on the phenotype and co-expression, we conclude that *alx-3* is most likely to be required cell-autonomously in regenerating *notum*^+^ pole precursor cells.

Muscle cells play an important role in the expression of PCGs, thus we assessed the role of *alx-3* in maintaining the muscle cell population. *alx-3* is expressed in a subset of *collagen^+^* muscle cells (11±2.3%) at anterior-facing wound sites at 24 HPA (Fig. S9A), and we assessed for any changes in body wall musculature in *alx-3(RNAi)* animals during regeneration. Fluorescence *in situ* hybridization for *collagen* revealed no significant difference in the proportion of *collagen*^+^ cells in *alx-3(RNAi)* tail fragments at 72 HPA or in intact animals compared with *control(RNAi)* (Fig. S9B and S9C, respectively). Similarly, there was no substantial difference in muscle fiber morphology or density in *alx-3(RNAi)* tail fragments at 72 HPA or in intact animals compared with *control(RNAi)*, observed using 6G10 immunostaining labelling muscle fibers (Fig. S9D and S9E, respectively). Together, these negative data indicate that although *alx-3* is expressed in a subset of muscle cells and *alx-3(RNAi)* results in dysregulated PCG expression occurring in muscle cells, this is not due to a reduction in the muscle cell population or to disrupted muscle fiber morphology.

To investigate whether Wnt signaling feeds back on wound-induced *alx-3* expression, knockdown of *β-catenin*, *APC* or *notum* were performed followed by post-pharyngeal amputation and whole-mount *in situ* hybridization of *alx-3* to assess wound-induced *alx-3* expression 48 HPA (Fig. 7C). Knockdown of *β-catenin* inhibits Wnt signaling, which resulted in higher expression of *alx-3* at anterior facing wounds, as well as ectopic *alx-3* expression near posterior regions of tail fragments associated with ectopic brain tissue. In contrast, knockdown of *APC* or *notum*, which results in higher Wnt signaling, significantly reduced wound-induced *alx-3* expression at anterior-facing wounds by whole-mount *in situ* hybridization (Fig. 7C) and qRT-PCR (Fig. S10).

Owing to the feedback between Wnt signaling and *alx-3*, we reasoned that a low-Wnt environment in *β-catenin(RNAi)* may be able to rescue the headless phenotype of *alx-3(RNAi)* tail fragments. To test this, we performed double RNAi using *β-catenin(RNAi)* with *alx-3(RNAi).* Knocking down *β-catenin* in tail fragments resulted in anterior brain regeneration similar to controls visualized by fluorescence *in situ* hybridization with probes against *pc2* and *gpas*. Double knockdown of *β-catenin* with *alx-3* resulted in a significantly higher proportion of tail fragments that were able to regenerate a brain [93% compared with 53% in *alx-3(RNAi)* animals; Fig. 7D]. Interestingly, knockdown of *β-catenin* is already known to ablate wound-induced *notum* expression, and knocking down both *β-catenin* and *alx-3* did not recue *notum* expression 24 HPA (Petersen and Reddien, 2011) (Fig. 7E). However, knockdown of *APC* increases wound-induced *alx-3* expression, and knocking down both *alx-3* and *APC* rescued this phenotype, Therefore, rescue of the *alx-3(RNAi)* phenotype when *β-catenin* expression is depleted occurs through a mechanism that does not involve rescuing wound-induced *notum* expression. Taken together, the rescue of the *alx-3(RNAi)* phenotype by inhibition of Wnt signaling suggests that *alx-3* is in a negative-feedback loop regulated by Wnt signaling.

## DISCUSSION

In this study, we functionally tested the *aristaless-like homeobox-3* (*alx-3*) transcription factor that was originally found to be expressed in a subpopulation of dividing *piwi-1*^+^ cells called ν-neoblasts (Molinaro and Pearson, 2016). We found that *alx-3* was required to maintain *gad^+^* and *th^+^* neurons as well as maintain brain size in proportion to body size in uninjured animals. We expected more specific effects on regenerating neurons, but were surprised to find that *alx-3* was induced in stem cells located at anterior-facing wounds during regeneration, and that tail fragments could no longer regenerate heads. Defects in head regeneration could be due to failure of neurogenesis or to a defect in re-establishing anterior polarity, thereby leading to a failure in regeneration of all anterior structures. To distinguish which of these mechanisms were affected, we performed sagittal amputations to remove neural tissue while maintaining the AP axis. *alx-3(RNAi)* animals that maintained full AP polarity were able to regenerate missing neural tissue, suggesting the regeneration defect was not due to failure of neurogenesis specifically. We then used bulk RNAseq to uncover the pathways that were dysregulated in regenerating *alx-3(RNAi)* tails. We found that the *alx-3* headless tail phenotype was the result of an overall failure to initiate the early wound response transcriptional programs involving both *wnt1* and *notum*. The effect of early-acting wound response gene expression on *wnt1* and *notum* expression is currently unclear as knockdown of several of these early-acting genes failed to produce significant regeneration phenotypes (Wenemoser et al., 2012). This would suggest that multiple early wound response genes have redundant function to activate downstream *wnt1* and *notum* expression, and that *alx-3* may be one of the early factors to activate *wnt1* and *notum* either directly or indirectly. However, this hypothesis would be difficult to test, as we are unable to inhibit the expression of all early-acting genes involved in this wound response simultaneously. As PCGs, including *wnt1* and *notum*, are expressed in muscle cells, we investigated whether reduced expression of *wnt1* and *notum* in *alx-3(RNAi)* animals is due to dysregulation of the muscle population. However, we did not observe any significant difference in the proportion of *collagen^+^* muscle cells nor any perturbation in muscle fiber morphology in *alx-3*-depleted animals during regeneration or in uninjured animals. This suggests that *alx-3(RNAi)* inhibits *wnt1* and *notum* expression without affecting the number or morphology of muscle cells.

It is known that *notum* expression at anterior-facing wounds is crucial for establishing anterior regeneration polarity. Because knockdown of either *alx-3* or *notum* results in impaired head regeneration, this suggests that *alx-3* promotes *notum* expression in muscle cells. We put forth a model (Fig. 7F) that *alx-3* activates *notum* expression, either directly or indirectly, in muscle cells in a cell-autonomous manner. Expression of *notum* is then transmitted to neighboring stem cells at anterior-facing wound sites to subsequently activate *alx-3* expression cell non-autonomously to facilitate anterior regeneration.

Neoblasts are a pluripotent class of stem cells as they are constitutively active and give rise to all missing tissues during regeneration. Previous work has shown that at least some stem cells express Wnt signaling receptors, suggesting that they are capable of receiving Wnt signaling at homeostasis (Currie et al., 2016). The expression of *alx-3* within stem cells at anterior-facing wounds serves as a distinguishing factor between stem cells located at anterior-facing versus posterior-facing wounds. Before this work, *notum* expression was considered the primary differentiating factor between anterior-facing and posterior-facing wounds, although much of its expression is restricted to *collagen^+^* muscle cells (Scimone et al., 2017; Witchley et al., 2013). The regulation of *alx-3* expression in stem cells by known PCGs serves as an important relationship between positional signaling that is generally restricted to muscle cells and stem cells to guide cell fate and the regenerative outcome.

### A conserved role for *alx-3* in anterior pattering

*alx-3* is an aristaless-like paired homeobox transcription factor (Qian et al., 1992). Mice and humans have three Alx genes: *Alx1*, *Alx3* and *Alx4*; *Alx3* has been lost independently in some vertebrate lineages, including frogs (*X. tropicalis*), lizards (*A. carolinensis*) and chickens (*G. gallus*) (McGonnell et al., 2011)*.* Although Alx genes derived their names from the *Drosophila melanogaster* homolog *aristaless*, Alx-like genes are present across the metazoans (McGonnell et al., 2011; Ryan et al., 2006). The mouse *Alx3* homolog is expressed in the cephalic mesenchyme and lateral mesoderm, and is developmentally important for neural tube closure and craniofacial development (Beverdam et al., 2001; Lakhwani et al., 2010). In zebrafish, *Alx3* has a well-established role in craniofacial development: *Alx3* is enriched in frontonasal neural crest cells, and loss of *Alx3* results in severe neurocranium development defects (Mitchell et al., 2021; Yoon et al., 2022). In humans, homozygous mutation of *Alx3* has been associated with a form of frontonasal dysplasia known as frontorhiny (Twigg et al., 2009). Across several organisms, and including this study, *alx-3* plays an important role in establishing tissue identity and in the development of anterior structures, perhaps indicating that the role of *alx-3* in anterior tissue patterning in planarians may be a conserved function of this gene family. It will be interesting to determine whether Alx genes are upstream of *notum* and Wnt signaling in other systems during embryonic development.

## MATERIALS AND METHODS

### Animal husbandry and RNAi

Asexual populations of *Schmidtea mediterranea* (strain CIW4) were maintained as previously described (Alvarado et al., 2002). Planarians were kept at 18°C in milliQ water supplemented with 0.21 g/l Instant Ocean, 0.1 mM KCl, 0.1 mM MgSO_4_ and 0.12 mM NaHCO_3_. Animals were fed calf liver paste approximately once per week.

### Cloning and RNA interference (RNAi)

Planarian transcripts were cloned using forward and reverse primers to generate double-stranded RNA (dsRNA) from a pT4P expression plasmid. Cloning of *alx-3* was performed using an expression vector containing the *alx-3* gene (SmedASXL_006490, dd_Smed_v6_11150_0_1) (Rozanski et al., 2019) provided by Molinaro and Pearson (2016). All genes were cloned using forward and reverse primers into T4P vectors as previously described (Rink et al., 2009). Primers were generated using the freely available web tool Primer3 Web version 4.1.0 (https://primer3.ut.ee). To generate RNAi food, HT115 bacterial cultures expressing dsRNA were prepared using clones expressing the pT4P vector mixed with calf liver. RNAi experiments were performed as originally described (Newmark et al., 2003), with updated modifications (Adler and Alvarado, 2018). Briefly, bacteria were grown to an OD_600_ of 0.8 and induced with 1 mM isopropyl β-D-1-thiogalactopyranoside (IPTG) for 2 h at 37°C with shaking. Bacteria were pelleted and mixed with calf liver paste at a ratio of 500 μl of liver per 100 ml of original culture volume. Bacterial pellets were thoroughly mixed into the liver paste and frozen as aliquots at −80°C. GFP was used for all negative controls as previously described (Cowles et al., 2013). In all homeostatic experiments and regeneration experiments, RNAi food was fed to 7-day starved worms every third day for a total of nine feedings. In the homeostatic experiments, animals were fixed 10 days after the 9th feed. Amputations were performed 4 days after the 9th feed unless noted otherwise. All animals used for staining were 3-6 mm in length in the case of wild-type and homeostatic experiments, and 1-4 mm in the case of regenerating fragments, and were size matched between experimental and control worms.

### Riboprobe synthesis

Primer set AA18 (5′-CATTACCATCCCGCCACCGGTTCCATGG-3′) and PR244F (5′-GGCCCCAAGGGGTTATGTGG-3′) were used to generate a PCR template containing one T7 promoter site in the antisense direction of the gene of interest inserted in a pT4P vector as described previously (Currie et al., 2016). Antisense riboprobes were made using T7 RNA-polymerase (ThermoFisher Scientific, EP0111) over a 2-4 h incubation period. However, in the case of weakly expressed genes such as *alx-3*, an overnight incubation of the DIG probes resulted in optimal detection of the gene transcript.

### *In situ* hybridization, imaging and quantification

Riboprobes were made from PCR templates using the pT4P vector generated above. Whole-mount and fluorescent *in situ* hybridization experiments were performed as previously described (Currie et al., 2016; Pearson et al., 2009) with the following modifications that optimized results. Briefly, 5% N-acetylcysteine in phosphate-buffered saline (PBS) was used to kill the worms and remove mucus, followed by fixation in 4% formaldehyde in PBST (0.3% Triton-X) for 20 min. Worms were rinsed with PBST and further permeabilized with reduction solution consisting of 50 mM DTT, 1% NP-40 and 0.5% SDS in PBS for 3-5 min at room temperature. Worms were dehydrated with methanol and stored at −20°C then bleached with 6% hydrogen peroxide (in methanol) overnight and rehydrated with PBST. Worms were pre-hybridized for 2 h at 56°C then hybridized with probe overnight at 56°C. Blocking solution (10% horse serum in MABT) was used for blocking and antibody incubation. Colorimetric stains (whole-mount *in situ* hybridization) were developed using 4-nitro blue tetrazolium chloride (NBT, Roche, 11383213001) and 5-bromo-4-chloro-3-indolyl-phosphate (BCIP, Roche, 11383221001). Fluorescence *in situ* hybridization stains were developed with either tyramide amplification in borate buffer, or Fast Blue B salt (Sigma D9805) with naphthol AS-MX phosphate (Sigma 855). For immunostaining, rabbit anti-H3ser10p (H3P) was used at 1:1000 (EMD Millipore, 05-817R-I) or mouse anti-PIWI-1 (kind gift from Dr Jochen Rink, Max Planck Institute for Multidisciplinary Sciences, Germany) was used at 1:1000 (Zhu and Pearson, 2018). BrdU (Sigma-Aldrich B5002) labeling was performed as previously described (Zhu et al., 2015). Briefly, worms were adapted to a high salt medium (5 g/L Instant Ocean) for 1-3 days before BrdU delivery and maintained in high salt for the duration of the chase period. BrdU dissolved in 50% DMSO was fed at a concentration of 10 mg/ml in liver paste. Fixation and fluorescence *in situ* hybridization were performed as described above. After fluorescence *in situ* hybridization, worms were incubated in acid for 45 min (2 N HCl and 0.5% Triton-X), then neutralized with 0.1 M sodium borate. Blocking solution (10% BSA and 5 mM thymidine in PBST) was used for blocking and antibody incubation. BrdU was detected with mouse anti-BrdU at 1:300, followed by anti-mouse HRP (1:500) and tyramide amplification. Only animals that exhibited staining throughout the entire body were quantified.

### Microscopy, processing and analysis

Colorimetric whole-mount *in situ* hybridization stains were imaged on a Leica M165 fluorescent dissecting microscope. Fluorescent stains were imaged on a spinning disk confocal microscope (Olympus IX81S1F-3) with a Hamamatsu C9100-13 EM-CCD camera and a Yokogawa CSU X1 scan head, employing Perkin Elmer Volocity software. All image quantifications and post-processing were made using the freely available ImageJ software Fiji (http://rsb.info.nih.gov/ij/) and Imaris (http://www.bitplane.com/imaris/imaris). All experiments were, at minimum, triplicated and at least 10 worms were used per stain and per time point (i.e. *n*≥30). All statistical analyses between RNAi groups were carried out using a two-tailed Student's *t*-test. All images were post-processed using Adobe Photoshop and figures assembled in Inkscape.

### Quantitative real-time PCR

Reverse transcription reactions were conducted on total RNA extracted from ∼10 whole worms or 20 tail fragments using Trizol Reagent (Thermo Fisher) a SuperScript III Reverse Transcriptase Kit (Invitrogen). Quantitative real-time PCR was performed in biological triplicate on a Bio-Rad CFX96 Touch Real-Time PCR Detection System with SYBR Green PCR Master Mix (Roche) as per the manufacturer's instructions. Expression was normalized to *control(RNAi)* and the 2^−ΔΔCT^ method was used for relative quantification. Primer pairs for ubiquitously expressed GAPDH were used as a reference as previously described (Eisenhoffer et al., 2008). Experiments were carried out in biological and technical triplicates. All statistical analysis was carried out by performing Welch's unequal variances *t*-test using Microsoft Excel. *P<0.05, **P<0.01 and ***P<0.001. Graphs were generated using GraphPad Prism. All error bars indicate the s.d.

### RNA sequencing

RNA deep sequencing (RNAseq) was performed on whole and amputated tail fragments 4 days after nine RNAi feedings for *control*(*RNAi*) and *alx-3*(*RNAi*). RNA was extracted using Trizol RNA Extraction Kit (Thermo Fisher). Experiments were performed in biological triplicates and sequenced to a depth of ∼30 million reads per sample and multiplexed on an Illumina NovaSeq with 50 bp paired-end reads. Primer sequences were removed using trimmomatic and sequences were then aligned to a previously described planarian transcriptome Smed_ASXL in NCBI Bio Project PRJNA215411 using the program Salmon (https://bioconductor.org/packages/devel/workflows/vignettes/rnaseqDTU/inst/doc/rnaseqDTU.html; Love et al., 2018). DESeq2 (https://bioconductor.org/packages/release/bioc/html/DESeq2.html) (Love et al., 2014) was used to determine significantly up- or downregulated transcripts in *control(RNAi)* and *alx-3(RNAi)* animals. Heatmaps were generated using the pheatmap R package in RStudio. DESeq2 output on processed data for all time points are in Table S1.

## Supplementary Material

10.1242/develop.201777_sup1 Supplementary information
