# Supplementary material for: aristaless-like homeobox-3 is wound induced and promotes a low-Wnt environment required for planarian head regeneration
Source: Development. 2023 Sep 25;150(18):dev201777. doi: 10.1242/dev.201777 (PMC10560571; doi:10.1242/dev.201777)
Supplement: Supplementary information [file develop-150-201777-s1.pdf]

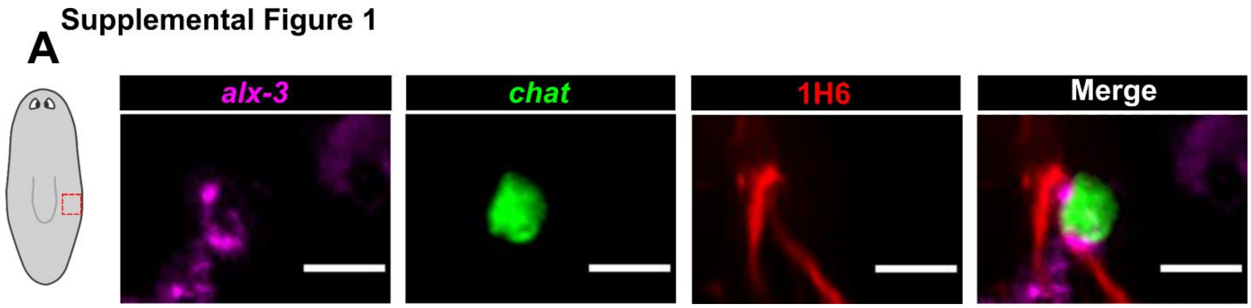

Fig. S1. *alx-3* is expressed in neurons around the periphery of the body.

FISH *alx-3* and *chat* expression and immunostaining of α-1H6 labeling neuronal axons in wildtype whole animals. Scale bars, 10µm.

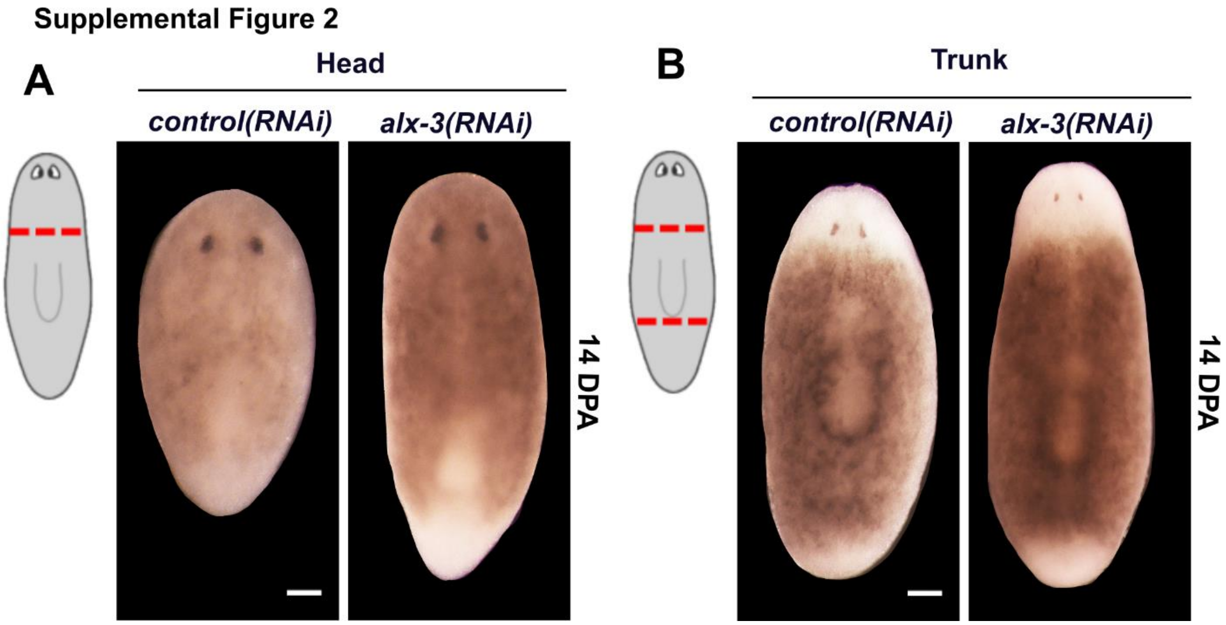

Fig. S2. *alx-3* is not required for regeneration of head and trunk fragments.

(A) Live images of *control(RNAi)* or *alx-3(RNAi)* head fragments after pre-pharyngeal transverse amputation and 14 days of regeneration. Scale bars, 100µm.

(B) Live images of *control(RNAi)* or *alx-3(RNAi)* trunk fragments after pre- and post-pharyngeal transverse amputation and 14 days of regeneration. Scale bars, 100µm.

Development • Supplementary information

Supplemental Figure 3

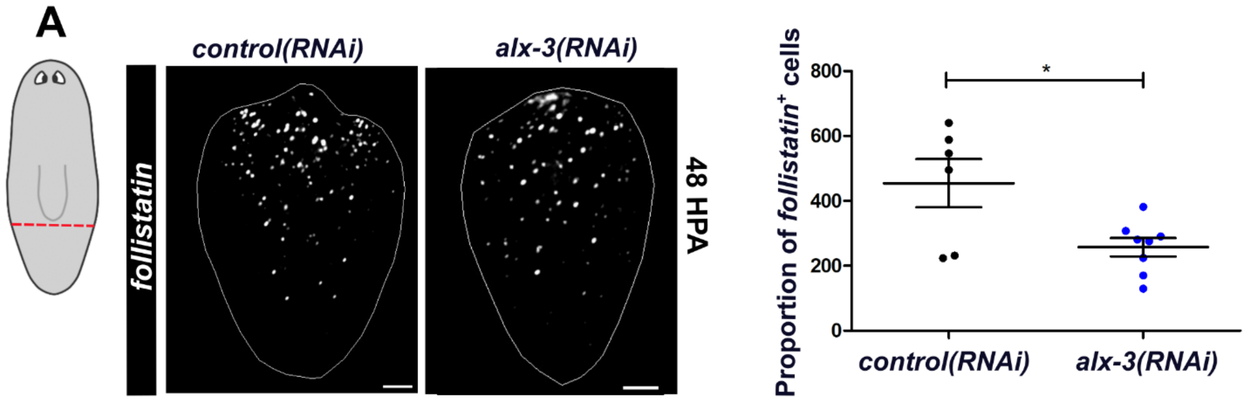

Fig. S3. *Follistatin* expression is reduced but not ablated in *alx-3(RNAi)* tail fragments at 48HPA

(A) FISH of *follistatin* at 48HPA in *alx-3(RNAi)* tail fragments 48HPA. Scale bars= 100µm. Quantification of proportion of cells that express *follistatin*. Quantifications are the mean ±1 S.D.

Supplemental Figure 4

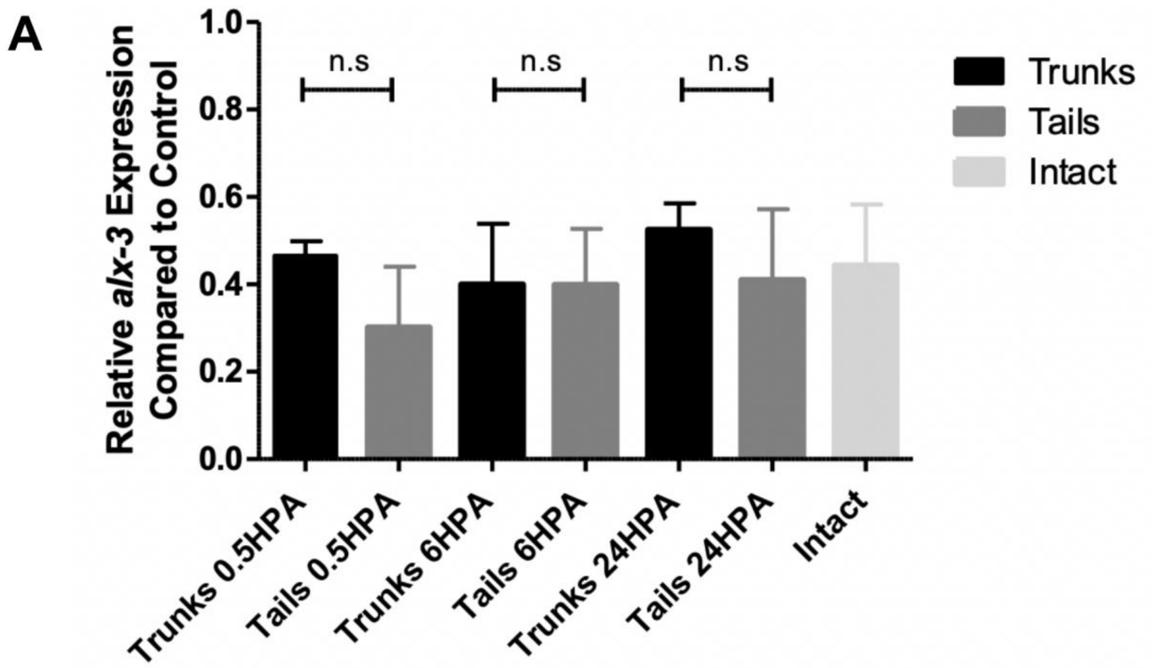

Fig. S4. Quantitative real-time PCR and WISH of alx-3expression in alx-3(RNAi) animals. qPCR quantification of alx-3 expression in wild-type tail and trunk fragments and intact animals relative to controls 0.5HPA, 6HPA, and 24 HPA and in uninjured animals. Quantifications are mean ±1 S.D

Development • Supplementary information

Supplemental Figure 5

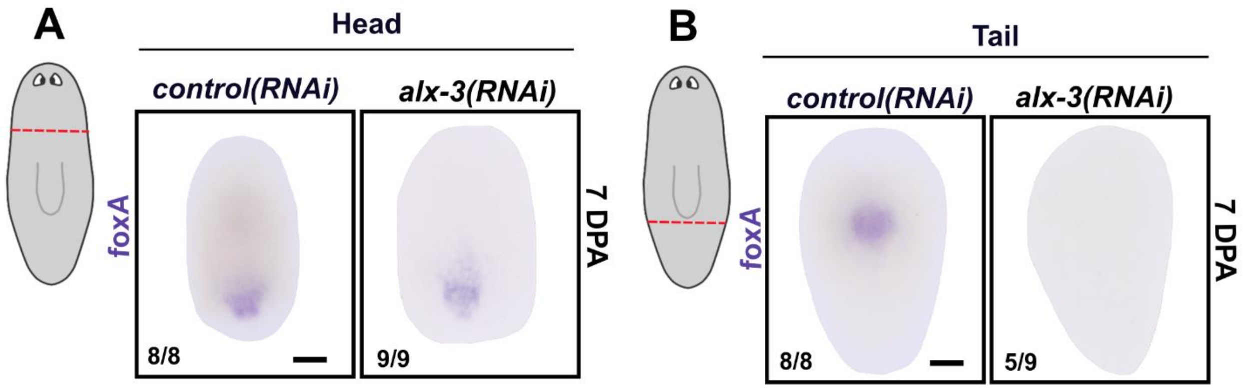

Fig. S5. *alx-3(RNAi)* tail fragments do not regenerate a pharynx.

(A) WISH of *foxA* in *alx-3(RNAi)* head fragments 7DPA. Scale bars= 100µm.

(B) WISH of *foxA* in *alx-3(RNAi)* tail fragments 7DPA. Scale bars= 100µm.

Development • Supplementary information

Supplemental Figure 6

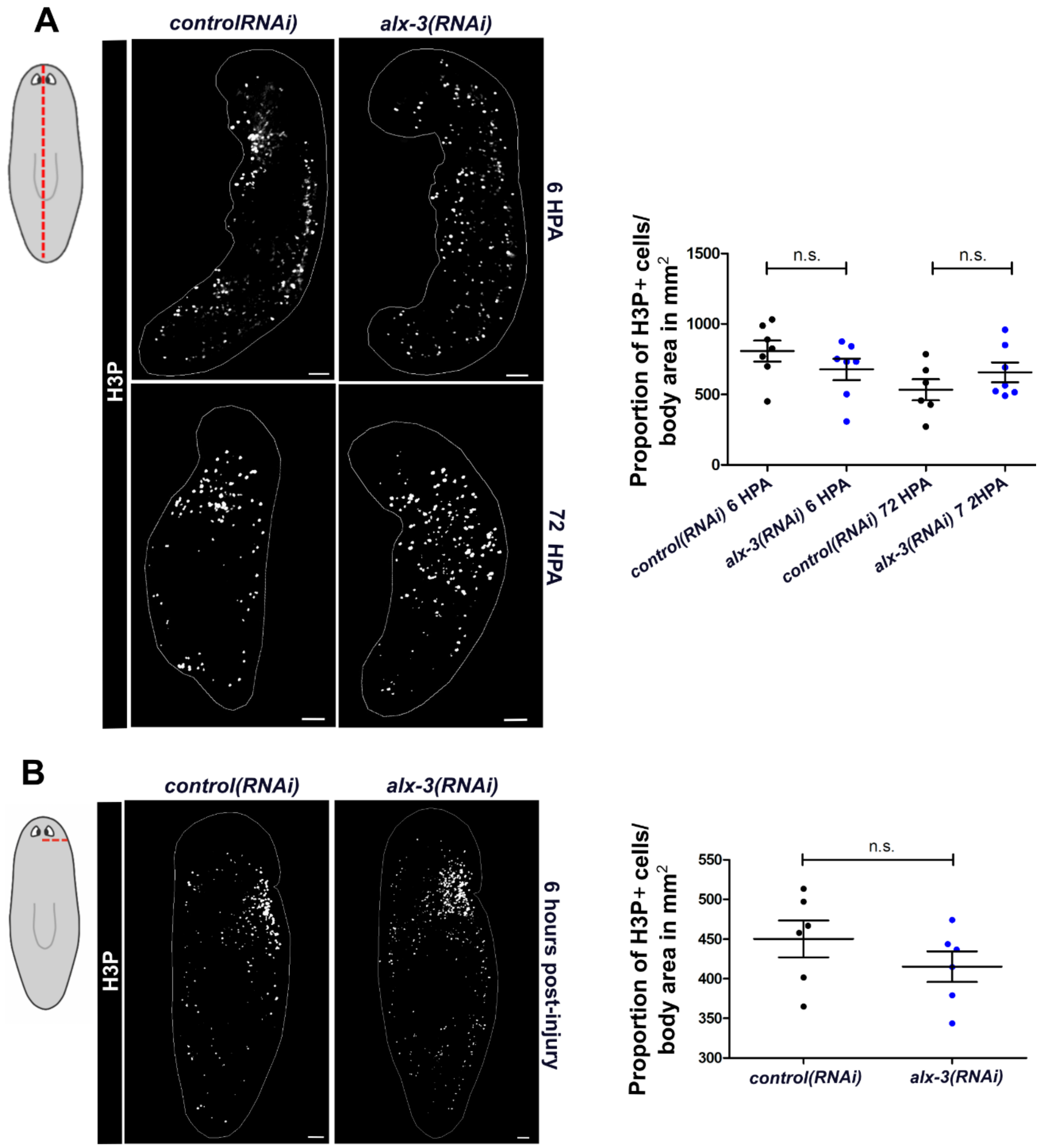

Fig. S6. *alx-3* knockdown does not affect stem cell proliferation following sagittal amputations or incision injuries.

(A) Immunostaining of α-H3P in sagittally-amputated *control(RNAi)* or *alx-3(RNAi)* animals at 6HPA or 72HPA, imaged ventrally. Quantification of the proportion of α-H3P+ cells normalized to area of the animal. Quantifications are the mean ±1 S.D. Scale bars = 100µm.

(B) Immunostaining of α-H3P in *control(RNAi)* or *alx-3(RNAi)* animals following a minor incision injury posterior to the right eye at 6 hours-post injury, imaged ventrally. Quantification of the proportion of α-H3P+ cells normalized to area of the animal. Quantifications are the mean ±1 S.D. Scale bars = 100µm.

Development • Supplementary information

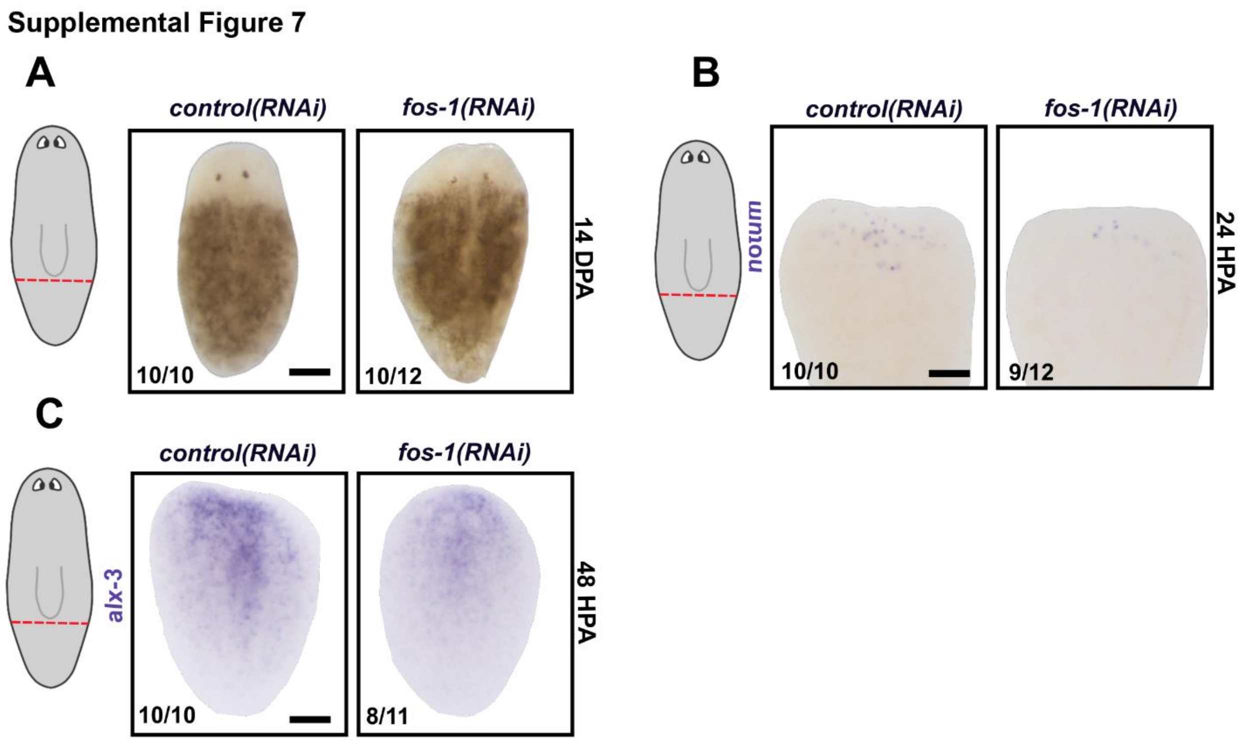

Fig. S7. *fos-1* RNAi animals exhibit reduced wound-induced *notum* and *alx-3* expression.

(A) Live image of *fos-1(RNAi)* tail fragments at 14DPA. Scale bars = 100µm.

(B) WISH for *notum* in *fos-1(RNAi)* tail fragments at 24HPA. Scale bars = 100µm.

(C) WISH of *alx-3* in f*os-1(RNAi)* tail fragments at 48HPA. Scale bars = 100µm.

Supplemental Figure 8

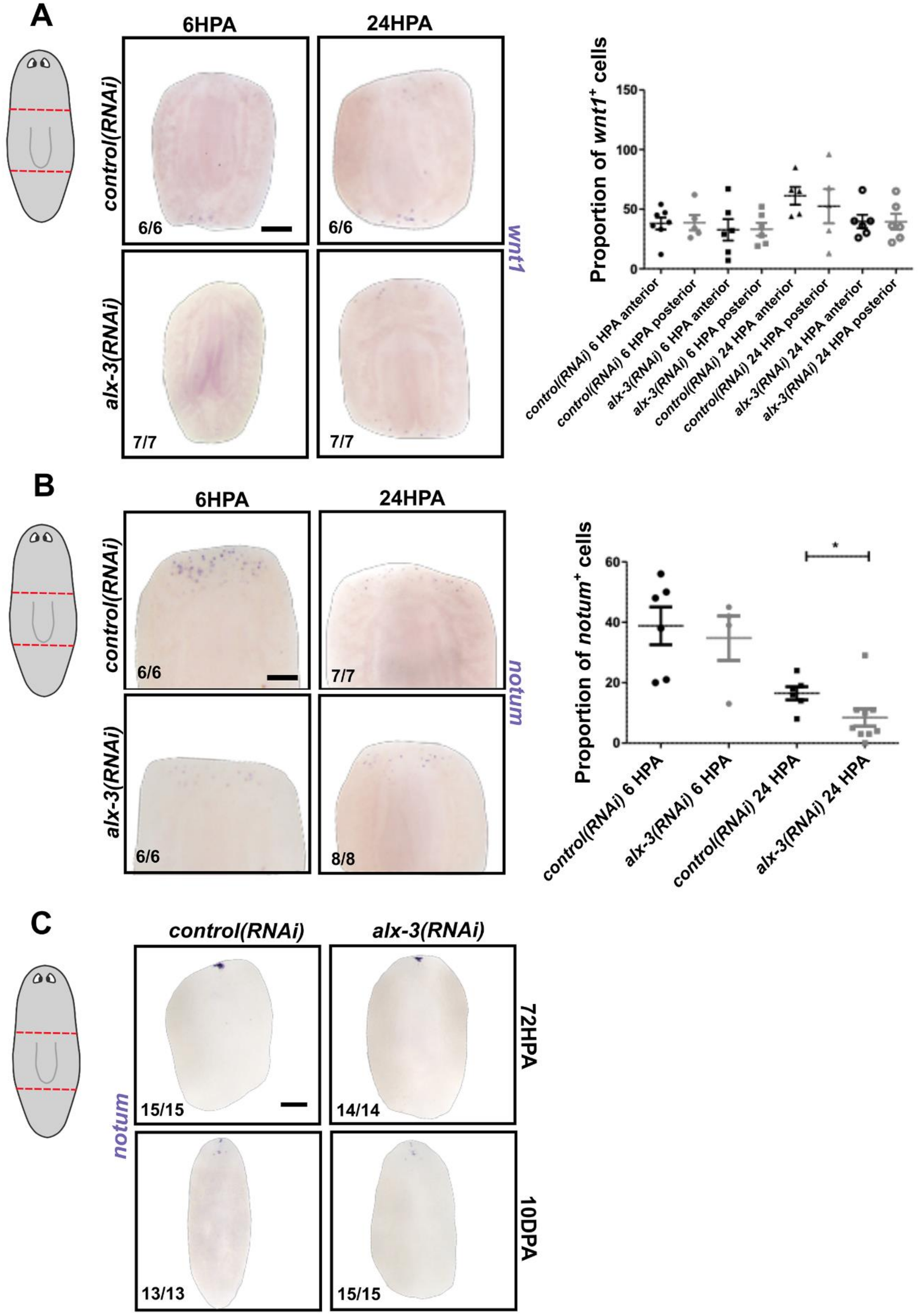

Development • Supplementary information

Fig. S8. Regulation of Wnt signaling by *alx-3* is not as prominent in trunk fragments.

(A) WISH for *wnt1* expression in *control(RNAi)* or *alx-3(RNAi)* trunk fragments at 6 and 24 HPA. Quantification of proportion of cells that express *wnt1* at anterior-facing and posterior-facing wound sites. Quantifications are the mean ±1 S.D

(B) WISH for *notum* expression in *control(RNAi)* or *alx-3(RNAi)* amputated trunk fragments at 6 or 24 HPA. Quantification of proportion of cells that express *notum* at anterior-facing wound sites. Quantifications are the mean ±1 S.D.

(C) WISH for *notum* expression in *control(RNAi)* or *alx-3(RNAi)* trunk fragments at 72 HPA or 10 DPA.

Development • Supplementary information

Supplemental Figure 9

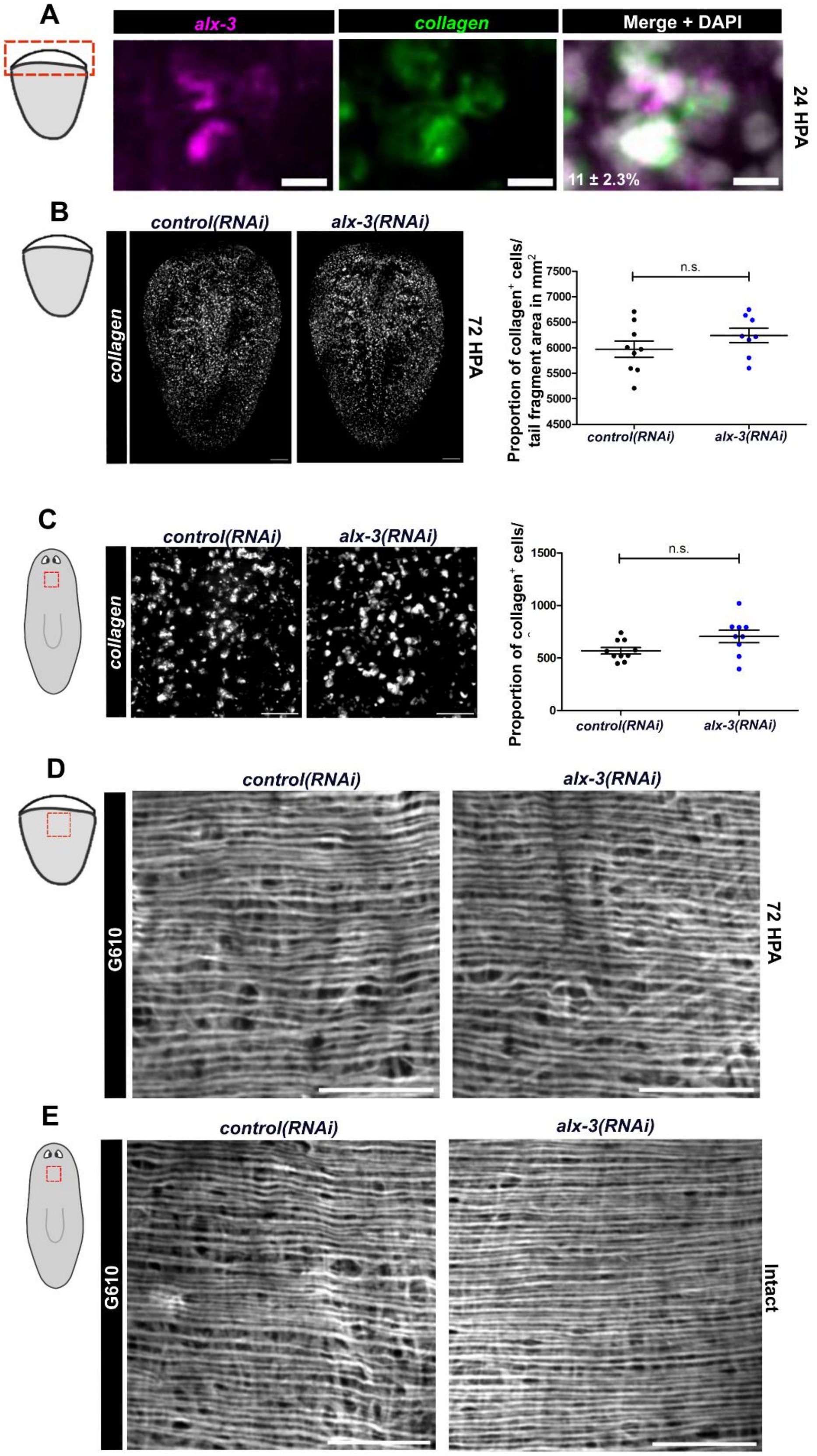

Development • Supplementary information

Fig. S9. *alx-3* is expressed in a subset of *collagen*$^+$ muscle cells at 24HPA but *alx-3(RNAi)* does not affect the proportion of *collagen*$^+$ cells or muscle fiber morphology.

(A) dFISH for *alx-3* and *collagen* at anterior-facing wound sites at 24HPA. Scale bars= 50µm.

(B) FISH for *collagen* in *alx-3(RNAi)* tail fragments at 72HPA. Scale bars= 50µm. Quantification of the proportion of cells that express *collagen* normalized to tail fragment area. Quantifications are the mean ±1 S.D.

(C) FISH for *collagen* in *alx-3(RNAi)* in intact animals. One square tile was imaged in the center of the animal posterior to the brain lobes. Scale bars= 50µm. Quantification of the proportion of cells that express *collagen* per image. Quantifications are the mean ±1 S.D.

(D) Immunofluorescent staining of body wall muscle using the monoclonal antibody 6G10 in *alx-3(RNAi)* tail fragments at 72HPA. Scale bars= 50µm.

(E) Immunofluorescent staining of body wall muscle using the monoclonal antibody 6G10 in *alx-3(RNAi)* intact animals. One square tile was imaged in the center of the animal posterior to the brain lobes. Scale bars= 50µm.

Development • Supplementary information

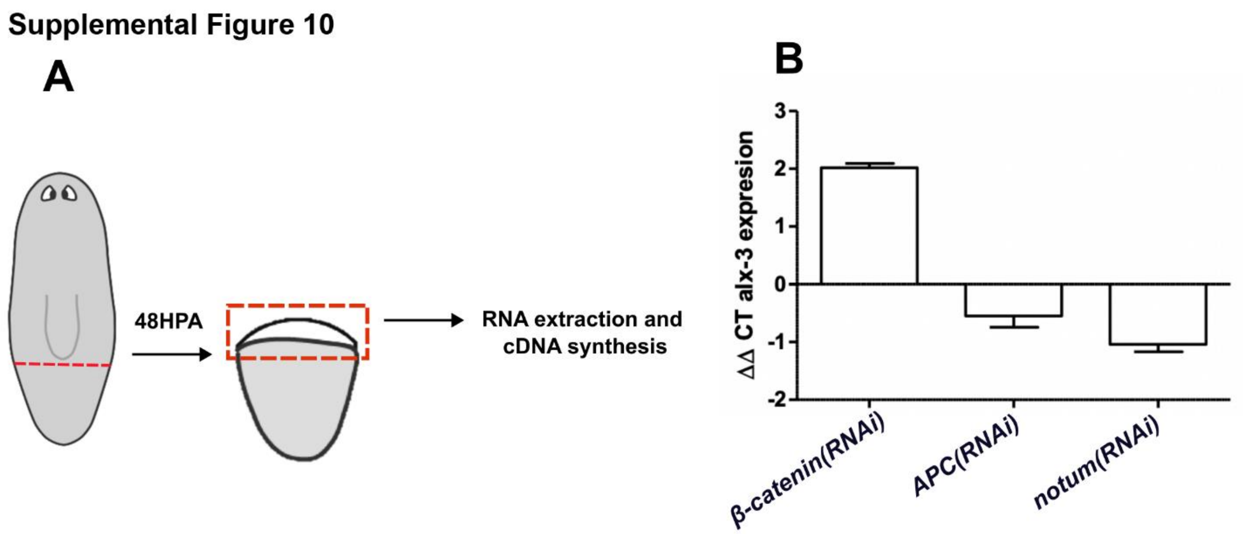

Fig. S10. qRT-PCR for *alx-3* at anterior-facing wound sites of *β-catenin, APC,* or *notum* RNAi tail fragments.

(A) Schematic of the qTR-PCR experiment. *control(RNAi), β-catenin(RNAi), APC(RNAi),* or *notum(RNAi)* animals were amputated posterior to the pharynx and regenerated for 48HPA. Approximately 0.5mm of tissue was collected from anterior-facing wound sites for RNA extraction and cDNA synthesis.

(B) Quantification of qRT-PCR displaying ΔΔCT values of *alx-3* expression, using GAPDH as the housekeeping gene.

Table S1. Processed RNAseq data from control or *alx-3* RNAi whole tail fragments during 72 h of regeneration

Click here to download Table S1
