# Supplementary material for: aristaless-like homeobox-3 is wound induced and promotes a low-Wnt environment required for planarian head regeneration
Source: Development. 2023 Sep 25;150(18):dev201777. doi: 10.1242/dev.201777 (PMC10560571; doi:10.1242/dev.201777)
Supplement: Supplementary file 1 [file develop-150-201777_review_history.pdf]

aristaless-like homeobox-3 is wound induced and promotes a low-Wnt environment required for planarian head regeneration

Zaleena Akheralie, Tanner J. Scidmore and Bret J. Pearson
DOI: 10.1242/dev.201777

Editor: Ken Poss

Review timeline
Original submission: 13 March 2023
Editorial decision: 12 April 2023
First revision received: 27 July 2023
Accepted: 30 August 2023

Original submission

First decision letter

MS ID#: DEVELOP/2023/201777

MS TITLE: aristaless-like homeobox-3 is wound induced in stem cells and required for planarian head regeneration

AUTHORS: Zaleena Akheralie, Tanner J. Scidmore, and Bret J. Pearson

I have now received all the referees' reports on the above manuscript, and have reached a decision. The referees' comments are appended below, or you can access them online: please go to BenchPress and click on the 'Manuscripts with Decisions' queue in the Author Area.

As you will see, the referees express considerable interest in your work, but have some significant criticisms and recommend a substantial revision of your manuscript before we can consider publication. If you are able to revise the manuscript along the lines suggested, which may involve further experiments, I will be happy receive a revised version of the manuscript. Your revised paper will be re-reviewed by one or more of the original referees, and acceptance of your manuscript will depend on your addressing satisfactorily the reviewers' major concerns. Please also note that Development will normally permit only one round of major revision. If it would be helpful, you are welcome to contact us to discuss your revision in greater detail. Please send us a point-by-point response indicating your plans for addressing the referee's comments, and we will look over this and provide further guidance.

Please attend to all of the reviewers' comments and ensure that you clearly highlight all changes made in the revised manuscript. Please avoid using 'Tracked changes' in Word files as these are lost in PDF conversion. I should be grateful if you would also provide a point-by-point response detailing how you have dealt with the points raised by the reviewers in the 'Response to Reviewers' box. If you do not agree with any of their criticisms or suggestions please explain clearly why this is so.

Reviewer 1

Advance summary and potential significance to field

This study describes the role of the aristaless-like transcription factor alx-3 in head regeneration in planarians. Planarians are a well-established model for the study of anterior-posterior polarity and

© 2023. Published by The Company of Biologists under the terms of the Creative Commons Attribution License (https://creativecommons.org/licenses/by/4.0/).

for tissue regeneration and this thus is a great model to examine the role of this transcription factor. The authors find that loss of alx-3 results in severe defects in head regeneration. This is not due to the inability to generate neuronal tissue, but they show evidence that this is related to the deregulation of the re-establishment of anterior polarity in an amputated tail piece, which is already detectable at 6 hours after amputation. They also find a reduction in cell proliferation in response to amputation and inability to establish a new anterior pole. Reduction of the levels of the posterior morphogen Wnt can rescue the alx-3(RNAi) defect in head regeneration. The authors interpret this to mean that there is feedback between Wnt signaling and alx-3 expression.

The manuscript is well-written, the assays are well executed, and the imaging is of high quality. I think this study presents a set of good data and will be of interest to the planarian community and to researchers interested in the regulation of tissue regeneration.

Comments for the author

The main limitation is that the manuscript remains descriptive as there is little definitive information in terms of mechanism. The authors start off by stating their central question: how are wound-induced programs positional identity programs, and neoblast proliferation programs integrated to ensure proper regeneration of specific structures? (Line 88-90) While this study does describe a transcription factor that affects both, it is unclear how much of the effects are direct, and how this would contribute to coordination. In particular, it remains unclear whether the effects on the stem cells are direct, or primarily through the failure of polarity. I could think of a few extra experiments that may tease this apart a bit further and clarify the relationship, but it will be difficult to fully resolve this. In the absence of more clarifying data alx-3 appears to be just another transcription factor that also contributes to new pole formation - as there have been several before and probably will be several more to be characterized.

1. The authors find that alx-3- tail pieces have reduced mitotic activity as well as absence of an anterior pole. It is somewhat surprising that the 1st mitotic wave is already affected as this does not typically rely on tissue absence or re-establishment of polarity. This could therefore be an important indication that the two effects are independent, but it would be helpful to strengthen this finding.

Were both mitotic waves also affected in the sagital cuts that regenerated fine and retained the anterior pole? And is the first mitotic wave also reduced during an incision when all polarity remains intact and no tissue as been removed?

2. The authors find that head regeneration in the absence of alx-3 is defective, but this is rescued by a concomitant reduction of b-catenin. The authors interpret this as evidence of a feed-back mechanism. However this could also be interpreted to mean that even in the absence of alx-3, a low Wnt environment is sufficient for head regeneration to proceed. The proposed increase in alx-3 levels upon b-catenin RNAi (Fig 7D), which would support the second half of the feedback loop, appears minimal to non-exisitent, unless there is better data available. The fact that half of the alx-3(RNAi) animals can still regenerate a head (line 195) also supports the idea that alx-3 assists in the reestablishment of anterior polarity, but is not essential. While a feedback loop is an interesting idea, it would require additional experiments to support this.

minor: The model in Figure 7F is a bit confusing on the molecular details. Notum is not a competitor of Wnt for receptor binding, but is a Wnt inactivator.

<u>Reviewer 2</u>

Advance summary and potential significance to field

In the manuscript ‘aristless-like homeobox-3 is wound-induced in stem cells and required for planarian head regeneration,’ Zaleena Akheralie and colleagues present significant data indicating that the planarian gene alx-3 is expressed in the planarian neural and muscle cell lineages, as well as evidence that alx-3 is required for maintenance of Dopaminergic and GABAergnic neurons at

© 2023. Published by The Company of Biologists under the terms of the Creative Commons Attribution License (https://creativecommons.org/licenses/by/4.0/).

homeostasis and regeneration of anterior tissues from posterior planarian tail fragments. Notably, in posterior tail fragments, alx-3 is required for wound-induced gene expression changes 6 hours post amputation (including anterior notum expression and Wnt1 expression) and the regeneration defects in these fragments can be rescued by depletion of beta-catenin. Together, these finding identify alx-3 as an important transcription factor mediating wound-induced induction of Wnt and Notum signaling components in anterior post-mitotic tissues and expansion progenitors required for anterior tissue regeneration. These results will clearly be of importance to the fields of regenerative and stem cell biology.

Comments for the author

The data presented by Akheralie et al. is clear, mostly convincing, and adequately quantified. The authors should also be commended on an excellent discussion. My major criticism is that the title and abstract do not reflect the authors' model that alx-3 is required for early wound response genes (most likely in muscle or epidermis). In fact, it sometimes feels as if the authors' narrative de-emphasizes the role alx-3 may play in muscle and epidermal cells in favor of stem cell and neural functions. Despite this, I believe that this is a highly valuable manuscript that would be of interest to the readers of Development. Prior to publication, the authors analysis/discussion should be revised to address these concerns. In addition, I have included a few minor experiments/controls that I think should or could be included to strengthen the authors' interpretations.

Major Criticisms:
- The title of the paper makes it seem like alx-3 primarily effects regeneration through stem cell function, but the data indicates that it likely plays a role in wound-induced gene expression outside of the stem cell compartment. Same for concluding sentence of the abstract.
- Figure 1 indicates that alx-3 is expressed in muscle progenitors and post-mitotic muscle cells. Since the body wall musculature plays such an important role in patterning (and wound-induced Wnt1/notum expression), why was alx-3 expression in muscle and its role in specifying circular, diagonal, longitudinal, or dorsal ventral muscle fibers not explored? The authors could determine if alx-3 co-localizes with muscle collagen and if alx-3 depletion alters muscle fiber organization. These results may prove more relevant to the regeneration phenotype than the neural maintenance data presented in Figure 2.
- Since the alx-3 depletion phenotype is dependent on the AP cut site (much like follistatin depletion phenotypes), it would be informative for the authors to determine if wound-induced follistatin expression is also impacted by alx-3 depletion and if follistatin inhibition reduces wound-induced alx-3 expression in stem cells (to complement the notum experiments in Figure 6B and 7D).
- It was not clear in the figures… are the tail fragments unable to regenerate any missing tissues, or just anterior neural structures? For example, is the pharynx regenerated? if so, is it in the right place?

Minor Criticism/Feedback:
- Since alx-3 depletion inhibits anterior notum expression in posterior tissues (stem-cell independent transcriptional changes) and the regeneration defects observed in posterior tail fragments are highly reminiscent of notum depletion, I wonder if the authors could perform any experiments that would help to clarify if alx-3-mediated changes in stem cells vs. epidermis/muscle are most responsible for the regeneration defects. Does beta-catenin depletion rescue wound-induced notum expression at 6 hours post amputation in alx-3 depleted animals? Along similar lines, can beta-catenin depletion rescue notum or depletion phenotypes to the same extent that it can rescue alx-3 depletion? The authors might also be able to use their comparison of ald-3 depleted trunk fragments (which can regenerate) and tail fragments (no regeneration) to explore this question.

© 2023. Published by The Company of Biologists under the terms of the Creative Commons Attribution License (https://creativecommons.org/licenses/by/4.0/).

First revision

Author response to reviewers' comments

We would like to thank the Reviewers for their helpful suggestions and overall positive review of our work. We have addressed all comments to the best of our ability and have included 6 additional supplemental figures. These new data and adding clarity to the text has undoubtedly made the manuscript much stronger. Below is a point-by-point response to any concerns, but the major changes to the manuscript are as follows:
Figures
- **Figure 7E: We included FISH of *notum* in *β-catenin(RNAi)* tail fragments at 24HPA, showing that *alx-3* and *β-catenin* double *RNAi* does not rescue *notum* depletion observed when *β-catenin* alone is knocked down. In contrast, *alx-3* and *APC* double knockdown suppresse the increased *notum* expression observed then *APC* alone is knocked down.**
- **Supplemental Figure 5 (new figure): WISH of the pharynx marker *foxA* showing that *alx-3(RNAi)* tail fragments that do not regenerate a head also do not regenerate a pharynx.**
- **Supplemental Figure 6 (new figure): We added new data showing that knocking down *alx-3* does not affect stem cell proliferation in sagittally-amputated animals at 6HPA or 72HPA, nor in animals with minor incision injuries 6HPA by performing immunostaining of H3P and quantifications.**
- **Supplemental Figure 7 (new figure): We investigated whether *fos-1* knockdown influenced regeneration, wound-induced *notum* expression, or *alx-3* expression after amputation (it did not).**
- **Supplemental Figure 9 (new figure): We assessed changes to body wall muscle cells in regenerating *alx-3(RNAi)* tail fragments. We used double FISH of *alx-3* and *collagen* showing that *alx-3* is co-expressed in a subset of muscle cells at anterior facing wound sites 24HPA. We also quantified the proportion of *collagen*$^+$ muscle cells in *alx-3(RNAi)* tail fragments compared to the control and determined if *alx-3(RNAi)* resulted in changes in muscle fiber morphology (it does not).**
- **Supplemental Figure 10 (new figure): We added qPCR data showing the extent of changes in *alx-3* expression in *β-catenin(RNAi)*, *APC(RNAi)*, or *notum(RNAi)* tail fragments that are shown in Figure 7C.**
- **Supplemental Figure 3 (new figure): We assessed whether *alx-3* regulated *follistatin* expression at 48HPA, and whether knocking down *follistatin* impacted wound-induced *alx-3* expression (we found positive results for both).**
- **Figure 7F: The model was altered to show that Notum inhibits Wnt binding to Wnt receptors, thus leading to reduced Wnt signaling in stem cells and increased *alx-3* expression**
- **In the text we have added in more about alx-3's role in muscle cells as asked, as well as added to the Discussion and heavily edited throughout for clarity.**

Reviewer 1 Advance Summary and Potential Significance to Field:
This study describes the role of the aristaless-like transcription factor alx-3 in head regeneration in planarians. Planarians are a well-established model for the study of anterior-posterior polarity and for tissue regeneration and this thus is a great model to examine the role of this transcription factor. The authors find that loss of alx-3 results in severe defects in head regeneration. This is not due to the inability to generate neuronal tissue, but they show evidence that this is related to the deregulation of the re-establishment of anterior polarity in an amputated tail piece, which is already detectable at 6 hours after amputation. They also find a reduction in cell proliferation in response to amputation and inability to establish a new anterior pole. Reduction of the levels of the posterior morphogen Wnt can rescue the alx-3(RNAi) defect in head regeneration. The authors interpret this to mean that there is feedback between Wnt signaling and alx-3 expression.

The manuscript is well-written, the assays are well executed, and the imaging is of high quality. I think this study presents a set of good data and will be of interest to the planarian community and to researchers interested in the regulation of tissue regeneration.

Reviewer 1 Comments for the Author:
The main limitation is that the manuscript remains descriptive as there is little definitive

© 2023. Published by The Company of Biologists under the terms of the Creative Commons Attribution License (https://creativecommons.org/licenses/by/4.0/).

information in terms of mechanism. The authors start off by stating their central question: how are wound-induced programs, positional identity programs, and neoblast proliferation programs integrated to ensure proper regeneration of specific structures? (Line 88-90) While this study does describe a transcription factor that affects both, it is unclear how much of the effects are direct, and how this would contribute to coordination. In particular, it remains unclear whether the effects on the stem cells are direct, or primarily through the failure of polarity. I could think of a few extra experiments that may tease this apart a bit further and clarify the relationship, but it will be difficult to fully resolve this. In the absence of more clarifying data alx-3 appears to be just another transcription factor that also contributes to new pole formation - as there have been several before and probably will be several more to be characterized.

We would like to thank the reviewer for their thoughtful feedback and suggestions. In order to tease apart a mechanism by which *alx-3* expression is regulated by early transcription factors that are activated in response to injury, we have investigated the expression of *fos-1* and its role on wound-induced *alx-3* expression and *notum* expression, and its overall role during regeneration. Similar to what was reported by Wenemoser et al., 2012, *fos-1(RNAi)* animals regenerated animals with small blastemas, and ultimately, these animals were able to regenerate anterior tissue. We noted that *fos-1(RNAi)* animals had less wound-induced *alx-3* and *notum* expression indicating that while *fos-1* regulates downstream wound response programs, as suggested by Wenemoser et al., 2012, it is likely that several early wound response transcription factors are redundant in function, therefore knocking down one gene may not significantly impact downstream wound response and regeneration. Furthermore, while we indicated that there is a stem cell-dependent role of *alx-3* to regenerate the anterior pole and activate stem cell proliferation in response to injury, we believe that there is also a stem cell-independent role of *alx-3* to activate early wound response genes as well as wound-induced *notum* and *wnt1* expression, which are known to not require stem cells. As mentioned, it is unclear whether the stem cell dependent role of *alx-3* is a result of the stem cell-independent role of *alx-3* and the resolution of planarians is limited in answering this question in a definitive manner. These new data mentioned above are in Supplemental Figure 7.

1. The authors find that alx-3- tail pieces have reduced mitotic activity as well as absence of an anterior pole. It is somewhat surprising that the 1st mitotic wave is already affected as this does not typically rely on tissue absence or re-establishment of polarity. This could therefore be an important indication that the two effects are independent, but it would be helpful to strengthen this finding.

Were both mitotic waves also affected in the sagital cuts that regenerated fine and retained the anterior pole? And is the first mitotic wave also reduced during an incision when all polarity remains intact and no tissue as been removed?
We thank the reviews for these suggestions. We agree that further assessment of the mitotic peaks would benefit our study. To address this, we performed immunostaining H3P in *alx-3(RNAi)* animals amputated sagittally. We found that both mitotic waves are not significantly disrupted. Furthermore, the first mitotic wave in *alx-3(RNAi)* animals with incision injuries is also not significantly disrupted. Together, this suggests that the role of *alx-3* on stem cell proliferation in response to injury is dependent on the disruption of anterior-posterior polarity, as observed with transverse amputations. The new H3P data are in Supplemental Figure 6.

2. The authors find that head regeneration in the absence of alx-3 is defective, but this is rescued by a concomitant reduction of b-catenin. The authors interpret this as evidence of a feed-back mechanism. However, this could also be interpreted to mean that even in the absence of alx-3, a low Wnt environment is sufficient for head regeneration to proceed. The proposed increase in alx-3 levels upon b-catenin RNAi (Fig 7D), which would support the second half of the feedback loop, appears minimal to non-exisitent, unless there is better data available. The fact that half of the alx-3(RNAi) animals can still regenerate a head (line 195) also supports the idea that alx-3 assists in the reestablishment of anterior polarity, but is not essential. While a feedback loop is an interesting idea, it would require additional experiments to support this.
Thank you for these suggestions. To address this, we performed qRT-PCR of anterior-facing wound sites of *alx-3(RNAi)* tail fragments at 48 HPA to quantify the reduction of *alx-3* expression in *APC(RNAi)* or *notum(RNAi)* animals and the increased expression in *β-catenin(RNAi)* animals. The new data are in Supplemental Figure 10.

© 2023. Published by The Company of Biologists under the terms of the Creative Commons Attribution License (https://creativecommons.org/licenses/by/4.0/).

We have shown that *alx-3* expression is necessary to induce *notum* expression after injury, and that knocking down *notum* expression ablates wound induced *alx-3* expression. We have also shown that knocking down *β-catenin*, increases wound-induced *alx-3* expression while knocking down *APC* reduces *alx-3* expression, To further support this, we performed FISH of *notum* at anterior-facing wound sites at 24 HPA in *β-catenin(RNAi)*, *APC(RNAi)* as well as in *β-catenin* and *alx-3* double RNAi or *APC* and *alx-3* double RNAi animals. Knocking down *β-catenin* ablates wound-induced *notum* expression and knocking down *APC* increases *notum* expression. Knocking down *alx-3* in *β-catenin(RNAi)* animals does not rescue *notum* expression while knocking down *alx-3* in *APC(RNAi)* reduces the *APC* phenotype. The new data have been added to Figure 7E. While it is difficult to definitively prove a feedback loop, we believe that the data presented sufficiently supports the idea that *alx-3* both upstream and downstream of Wnt signaling (*i.e.*, a feedback loop).

Minor:
The model in Figure 7F is a bit confusing on the molecular details. Notum is not a competitor of Wnt for receptor binding, but is a Wnt inactivator.

Thank you for the suggestion and we have adjusted Figure 7F to make more sense based on the known function of Notum in other systems.

Reviewer 2 Advance Summary and Potential Significance to Field:
In the manuscript 'aristless-like homeobox-3 is wound-induced in stem cells and required for planarian head regeneration,' Zaleena Akheralie and colleagues present significant data indicating that the planarian gene alx-3 is expressed in the planarian neural and muscle cell lineages, as well as evidence that alx-3 is required for maintenance of Dopaminergic and GABAergnic neurons at homeostasis and regeneration of anterior tissues from posterior planarian tail fragments. Notably, in posterior tail fragments, alx-3 is required for wound-induced gene expression changes 6 hours post amputation (including anterior notum expression and Wnt1 expression) and the regeneration defects in these fragments can be rescued by depletion of beta-catenin. Together, these finding identify alx-3 as an important transcription factor mediating wound-induced induction of Wnt and Notum signaling components in anterior post-mitotic tissues and expansion progenitors required for anterior tissue regeneration. These results will clearly be of importance to the fields of regenerative and stem cell biology.

Reviewer 2 Comments for the Author:
The data presented by Akheralie et al. is clear, mostly convincing, and adequately quantified. The authors should also be commended on an excellent discussion. My major criticism is that the title and abstract do not reflect the authors' model that alx-3 is required for early wound response genes (most likely in muscle or epidermis). In fact, it sometimes feels as if the authors' narrative de-emphasizes the role alx-3 may play in muscle and epidermal cells in favor of stem cell and neural functions. Despite this, I believe that this is a highly valuable manuscript that would be of interest to the readers of Development. Prior to publication, the authors analysis/discussion should be revised to address these concerns. In addition, I have included a few minor experiments/controls that I think should or could be included to strengthen the authors' interpretations.
We would like to thank the reviewer for their thoughtful comments and positive support of our work. Overall, we agree with this comment as the muscle population is important for early wound response genes and PCG expression. We have performed several sets of experiments and have reworked the text to further emphasize the role of *alx-3* in muscle cells. To test whether *alx-3* has other roles in muscle, we first performed dFISH of *alx-3* and *collagen* to determine whether *alx*-3 is expressed in *collagen*$^{+}$ muscle cells near anterior-facing wound sites. We also performed FISH of *collagen* to assess the proportion of *collagen*$^{+}$ cells in *alx-3(RNAi)* animals compared to controls in amputated tail fragments at 72 HPA as well as intact animals. We also performed immunostaining of the muscle fiber marker 6G10 to determine whether there were any gross morphological changes in *alx-3(RNAi)* animals compared to controls in amputated tail fragments at 72 HPA and intact animals. Overall, we do not observe significant effects of *alx-3(RNAi)* on muscle cells. These new data are in Supplemental Figure 9.

© 2023. Published by The Company of Biologists under the terms of the Creative Commons Attribution License (https://creativecommons.org/licenses/by/4.0/).

We also performed further investigation of the role of early wound response genes on activating *alx-3* expression in response to injury by performing WISH of *alx-3* in *fos-1(RNAi)* animals 48 HPA, when *alx-3* expression is expected at anterior-facing wound sites. These new data are in Supplemental Figure 7.

We have re-worked our abstract and results section to address these new data sections and to further emphasize expression of *alx-3* in muscle cells, its functional role in this population as well and its impact of the early wounding gene *fos-1*, to induce wound-induced *alx-3* expression. We believe that these data further support the role of *alx-3* in muscle cells to complement our work on its stem cells and neural functions.

Major Criticisms:
-The title of the paper makes it seem like alx-3 primarily effects regeneration through stem cell function, but the data indicates that it likely plays a role in wound-induced gene expression outside of the stem cell compartment. Same for concluding sentence of the abstract.
Thank you for these comments. We have adjusted the title and abstract to include more details on the role of *alx-3* in the muscle cell population in addition to its role in the stem cell population.

-Figure 1 indicates that alx-3 is expressed in muscle progenitors and post-mitotic muscle cells. Since the body wall musculature plays such an important role in patterning (and wound-induced Wnt1/notum expression), why was alx-3 expression in muscle and its role in specifying circular, diagonal, longitudinal, or dorsal ventral muscle fibers not explored? The authors could determine if alx-3 co-localizes with muscle collagen and if alx-3 depletion alters muscle fiber organization. These results may prove more relevant to the regeneration phenotype than the neural maintenance data presented in Figure 2.
Thank you for these suggestions. We performed FISH for *collagen* in *alx-3(RNAi)* tail fragments at 72HPA and quantified the proportion of *collagen*+ cells and found no significant reduction in *collagen*+ cell number. We then performed immunostaining using the monoclonal 6G10 to label muscle fibers in *alx-3(RNAi)* tail fragments to assess for gross morphological changes in muscle fibers and we did not detect any changes. These new data are in Supplemental Figure 9.

-Since the alx-3 depletion phenotype is dependent on the AP cut site (much like follistatin depletion phenotypes), it would be informative for the authors to determine if wound-induced follistatin expression is also impacted by alx-3 depletion and if follistatin inhibition reduces wound-induced alx-3 expression in stem cells (to complement the notum experiments in Figure 6B and 7D).

Thank you for these suggestions. We performed FISH for *follistatin* in *alx-3(RNAi)* tail fragments at 48HPA, which is when *follistatin* expression occurs at wounds sites. We observed a decrease in the proportion of *follistatin*$^{+}$ cells at anterior-facing wound sites of *alx-3(RNAi)*tail fragments compared to the control, but not an ablation of these cells. We also performed knockdown of *follistatin* and assessed wound-induced *alx-3* expression at 48HPA. We observed overall reduced *alx-3* expression in the knockdown of *follistatin*. These follistatin measurements are in Supplemental Figure 3. We could not fit the *follistatin* RNAi experiment into the text in a cohesive way, nor does it make sense to us, but here are the data.

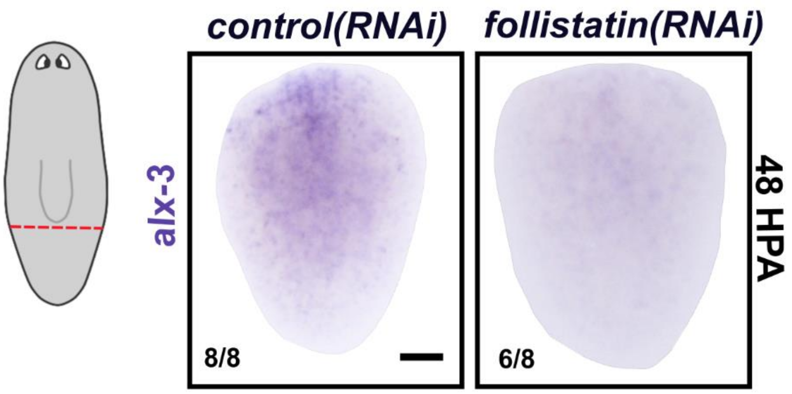

© 2023. Published by The Company of Biologists under the terms of the Creative Commons Attribution License (https://creativecommons.org/licenses/by/4.0/).

-It was not clear in the figures… are the tail fragments unable to regenerate any missing tissues, or just anterior neural structures? For example, is the pharynx regenerated? if so, is it in the right place?
To address the reviewer's question, we performed WISH of the pharynx marker *foxA* in *alx-3(RNAi)* tail fragments at 7DPA. This revealed that *alx-3(RNAi)* tail fragments that are unable to regenerate a head are also unable to regenerate other missing tissues, including a pharynx. These new data are presented in Supplemental Figure 5.

Minor Criticism/Feedback:
-Since alx-3 depletion inhibits anterior notum expression in posterior tissues (stem-cell independent transcriptional changes) and the regeneration defects observed in posterior tail fragments are highly reminiscent of notum depletion, I wonder if the authors could perform any experiments that would help to clarify if alx-3-mediated changes in stem cells vs. epidermis/muscle are most responsible for the regeneration defects. Does beta-catenin depletion rescue wound-induced notum expression at 6 hours post amputation in alx-3 depleted animals? Along similar lines, can beta-catenin depletion rescue notum or depletion phenotypes to the same extent that it can rescue alx-3 depletion? The authors might also be able to use their comparison of alx-3 depleted trunk fragments (which can regenerate) and tail fragments (no regeneration) to explore this question.
We greatly thank the reviewer for their intriguing questions. To address these questions, we performed FISH of *notum in alx-3* and *β-catenin* double RNAi as well as *alx-3* and *APC* double RNAi tail fragments at 24 HPA. It was previously known the depletion of *β-catenin* ablates wound-induced *notum* expression (Petersen and Reddien, 2011). Given this, we did not anticipate for depletion of both *β-catenin* and *alx-3* to rescue wound-induced notum expression, and it does not. In contrast, *APC(RNAi)* is known to increase *notum* expression at anterior-facing wounds at 24 HPA (Petersen and Reddien, 2011). We found that knocking down both *alx-3* and *APC* significantly reduces the proportion of *notum*$^+$ cells compared knocking down *APC* alone. These new data are in Figure 7E.

Second decision letter

MS ID#: DEVELOP/2023/201777

MS TITLE: aristaless-like homeobox-3 is wound-induced in muscle and stem cells and is required for planarian head regeneration

AUTHORS: Zaleena Akheralie, Tanner J Scidmore, and Bret J Pearson
ARTICLE TYPE: Research Article

I am happy to tell you that your manuscript has been accepted for publication in Development, pending our standard ethics checks. Reviewer #1 proposes a modification to the title of the paper, and I suggest that you consider this and adjust appropriately.

Reviewer 1

Advance summary and potential significance to field

(see previous round review)

Comments for the author

The authors should be commended on the additional work they did to address the reviewer questions. The increased emphasis on the effects of alx-3 on polarity and effects in the muscle benefits the clarity of the manuscript. This is a complicated topic with intricate regulatory interactions, and I think they have brought this as far as can be expected with the tools available in

© 2023. Published by The Company of Biologists under the terms of the Creative Commons Attribution License (https://creativecommons.org/licenses/by/4.0/).

the planarian field. In the future it will be interesting to find how exactly alx-3 ties in with (early) Wnt re-establishment and immediate early genes. For now however it is clear that alx-3 is involved in these processes, and this is a very nice contribution to the regeneration literature.

My only remaining comment is that to me the title (“aristaless-like homeobox-3 is wound-induced in muscle and stem cells and is required for planarian head regeneration) still does not reflect the content very well. I think the fact that head regeneration proceeds fine in the sagital amputations and the trunk fragments (as well as in half of the tails) suggests that alx-3 is not strictly “required” for the “regeneration” part, but rather facilitates it in hostile (high Wnt) context. It may be more accurate to say that alx-3 is important for the re-establishment of anterior polarity - and without that of course head regeneration is not possible. I understand though that even that is not a perfect description, and see the benefit of a shorter title, so I will leave it up to authors to consider.

<u>Reviewer 2</u>

Advance summary and potential significance to field

In the revised manuscript ‘aristless-like homeobox-3 is wound-induced in muscle and stem cells and is required for planarian head regeneration,’ Zaleena Akheralie and colleagues present significant and improved data indicating that the planarian gene alx-3 is expressed in stem cells, as well as the planarian neural and muscle cell lineages. Moreover, they provide evidence that alx-3 is required for maintenance of Dopaminergic and GABAergnic neurons at homeostasis and regeneration of missing tissues in posterior planarian tail fragments. Some significant improvements to the manuscript include expanded characterization of the alx-3/beta-catenin double knockdown and more detailed characterization of alx-3 regeneration and injury phenotypes (pharynx regeneration and wound-induced proliferation rates for non-transverse cuts). These new data are well executed, with high quality images and quantitation. While the main figures have not been significantly changed, the authors have addressed most of my previous concerns in the supplemental figures and I appreciate their effort to address my questions, even outside of the general narrative of the work (e.g. follistatin expression and muscle morphology). Finally, they have significantly re-written their discussion and title to acknowledge a role for alx-3 in wound-induced transcription both inside and outside of the stem cell compartment. I believe that this is a valuable and improved manuscript that would be of interest to the readers of Development. While there remain several interesting and important questions surrounding how alx-3 may regulate wound-induced gene expression in planaria, experiments to investigate this may be beyond the scope of this study. Therefore, I recommend acceptance with no further significant revisions beyond additional proof-reading.

Comments for the author

Typo in line 205: replace 'preformed' with performed

© 2023. Published by The Company of Biologists under the terms of the Creative Commons Attribution License (https://creativecommons.org/licenses/by/4.0/).
